# Aging-Related Moderation of the Link Between Compliance With International Physical Activity Recommendations and the Hemodynamic, Structural, and Functional Arterial Status of 3,619 Subjects Aged 3–90 Years

**DOI:** 10.3389/fspor.2022.800249

**Published:** 2022-02-21

**Authors:** Yanina Zócalo, Mariana Gómez-García, Juan Torrado, Daniel Bia

**Affiliations:** ^1^Departamento de Fisiología, Facultad de Medicina, Centro Universitario de Investigación, Innovación y Diagnóstico Arterial, Universidad de la República, Montevideo, Uruguay; ^2^CUiiDARTE - Movimiento, Actividad, Salud (CUiiDARTE-MAS), Comisión Sectorial de Investigación Científica, Universidad de la República, Montevideo, Uruguay; ^3^Departamento de Educación Física y Salud, Instituto Superior de Educación Física, Universidad de la República, Montevideo, Uruguay; ^4^Department of Internal Medicine, Jacobi Medical Center, Albert Einstein College of Medicine, New York, NY, United States

**Keywords:** adolescents, adults, arterial stiffness, central aortic pressure, children, intima-media thickness, physical activity recommendations, vascular reactivity (flow-mediated dilation)

## Abstract

**Background:**

Compliance with physical activity recommendations (CPARs) is associated with better health indicators. However, there are only few studies to date that have comprehensively analyzed the association between CPARs and cardiovascular status “as a whole” (e.g., analyzing hemodynamic, structural, and functional properties, and different arterial territories). The relationship between CPARs and cardiovascular properties could be strongly influenced by the growth and aging process.

**Aim:**

The goal of the study is to investigate the association between CPAR and cardiovascular properties by placing special emphasis on: (i) identifying if there is an independent association, (ii) if the association is “moderated” by age, and (iii) to what extent the association depends on the arterial parameter (hemodynamic vs. structural vs. functional) and/or the arterial segment (e.g., central vs. peripheral; elastic vs. transitional vs. muscular arteries).

**Methods:**

A total of 3,619 subjects (3–90 years of age) were studied. Extensive cardiovascular evaluations were performed. Cardiovascular risk factors (CRFs) and physical activity (PA) levels were determined. The subjects were categorized as compliant (*n* = 1, 969) or non-compliant (*n* = 1,650) with World Health Organization-related PA recommendations. Correlation and multiple regression models (including CPAR^*^Age interaction) were obtained, and Johnson-Neyman technique was used to produce regions of significance.

**Results:**

The independent association between CPARs and cardiovascular characteristics were strongly moderated by age. The moderation was observed on a wide range of age but particularly notorious on the extremes of life. Certain arterial characteristics demonstrated opposite effects in relation to CPAR status depending on the range of age considered. The association between CPAR and cardiovascular characteristics was independent of CRFs and moderated by age. In subjects younger than 45–55 years, CPAR status was associated with lower central and peripheral blood pressure (i.e., the younger the subject, the higher the reduction). During adult life, as age increases in the subjects, CPARs was associated with a beneficial hemodynamic profile, which is not related with variations in pressure but strongly related with lower levels of waveform-derived indexes and ventricular afterload determinants.

**Conclusions:**

The independent associations between CPARs and arterial properties were strongly moderated by age. Data provided by blood pressure levels and waveform-derived indexes would be enough to evaluate the independent association between CPARs and the vascular system in the general population.

## Introduction

Benefits of physical activity (PA) on quality of life, wellbeing, and overall body and mental health have been recently emphasized by the World Health Organization (WHO) in their latest global “age-specific” recommendations on PA for health (World Health Organization, 2019, 2020; Bull et al., 2020). According to these recommendations, while adults should undertake 150–300 min of moderate-intensity or 75–150 min of vigorous-intensity PA, or some equivalent combination of moderate-intensity and vigorous-intensity aerobic PA per week, an average of 60 min/day of moderate-to-vigorous intensity aerobic PA across the week is recommended for children and adolescents (Bull et al., 2020). Compliance with WHO guideline recommendations (“umbrals”) is associated with better “critical health indicators” such as lower adiposity and cardiometabolic biomarkers, and better physical fitness, motor skills, emotional regulation, self-esteem, cognition, and bone density (Saunders et al., 2016). However, whether compliance with PA recommendations (CPARs) is independently associated with benefits on some characteristics of the cardiovascular system, regardless of the age of the subject, is still a matter of debate.

At least three aspects remain to be evaluated in greater depth. First, the “beneficial effects” of PA on the cardiovascular system were initially thought to be associated with changes (improvement) in traditional cardiovascular risk factors (CRFs). However, a favorable CRF profile could only explain about half of the cardiovascular risk reduction associated with active lifestyle (Mora et al., 2007). As an example, although controversial, PA has been associated with reductions in blood pressure (BP) and arterial stiffness levels. However, arterial stiffness is not only determined by an “extrinsic” passive determinant associated with effects of BP levels on arterial wall distension (stretching), but also by an “intrinsic” component (e.g., elastin and collagen fiber stiffness and vascular smooth muscle stiffness and tone) (Bia et al., 2003). Consequently, the effects of CPARs on arterial stiffness could imply more than simple BP reduction (modification of a traditional CRF) but also effects on intrinsic properties of the arterial wall.

Second, PA levels have shown variable strengths of association with different vascular parameters (e.g., hemodynamic vs. structural vs. functional) and territories (e.g., elastic vs. muscular vs. transitional arteries, central vs. peripheral vessels) (Bia et al., 2009; Gómez-García et al., 2021). For instance, using PA questionnaires on a large population of children, we have shown that while PA components and subcomponents were significantly associated with both BP and structural arterial parameters, PA levels were not independently associated with regional arterial stiffness levels (Gómez-García et al., 2021). Similarly, we have also observed that the impact of several CRFs, such as obesity (Zócalo et al., 2017; Garcia-Espinosa et al., 2018), low birth weight and/or catch-up growth (Castro et al., 2019, 2021), and high BP (Zocalo et al., 2018), on the cardiovascular system also differs depending on the vascular parameter and/or the arterial territory considered. Based on these observations, it could be postulated that similar findings are expected with the association between CPARs and vascular properties throughout life. However, to our knowledge, there are no studies to date that have comprehensively analyzed the association between CPARs and cardiovascular status, considering the evaluation of the cardiovascular system “as a whole,” that is to say, analyzing the (i) global hemodynamic variables (e.g., cardiac output, systemic vascular resistances), (ii) peripheral and central measurements (e.g., brachial, tibial, and aortic BP levels), (iii) wave reflection contribution to recorded BP, (iv) structural (e.g., intima-media thickness and diameters) and functional arterial properties (e.g., local and regional arterial stiffness, macro- and micro-vascular reactivity indexes), and different vascular territories (e.g., elastic [carotid], transitional [brachial], and muscular [femoral] arteries) of same subjects.

Third, the relationship between CPARs and the hemodynamic, structural, and functional states of the arterial system could be strongly influenced by the aging process. A seminal study has shown that age can moderate the relationship among traditional CRFs and their impact on the arterial system (McEniery et al., 2010). In this sense, effects of PA on the cardiovascular system might also be modulated by age. This hypothesis has been tested in previous studies in which the relationship between PA and cardiovascular properties (e.g., BP, wave reflections, and/or arterial stiffness) differed significantly between different adult age range groups [e.g., <30 years [y], vs. >50 y (McDonnell et al., 2013), <45 vs. >45 y (Majerczak et al., 2019), 18–37 vs. 38–57 vs. 58–77 y (Tanaka et al., 2000), and 18–22 vs. 40–78 y] (Holland et al., 2017). For instance, one of these reports pointed out that the “greatest impact” or beneficial effects of PA on the cardiovascular system were observed in smaller pre-resistance and resistance vessels in younger subjects, and in large elastic arteries in older subjects (McDonnell et al., 2013). However, we are not aware of any study that has evaluated the association between CPARs and the arterial system considering large samples of children, adolescents, adults, and the elderly as a continuum, that is, considering practically the entire human lifespan (e.g., 3–90 y) without the need to build (and compare) “artificial” age range groups and performing a comprehensive assessment of the arterial system.

In this context, by studying a large sample of children, adolescents, adults, and the elderly, this study sought to investigate the association between CPARs and characteristics of the vascular system by placing a special emphasis on (i) identifying if there is independent association, (ii) if the association is “moderated” (influenced) by the aging process, and (iii) to what extent the association depends on the arterial parameter (hemodynamic vs. structural vs. functional) and/or the arterial segment (e.g., central vs. peripheral; elastic vs. transitional vs. muscular arteries) evaluated. For this purpose, a population study was carried out, in which 3,619 subjects between 3 and 90 y were studied by performing the same comprehensive non-invasive cardiovascular evaluation in each of the individual subjects.

## Methods

### Study Population

This study was carried out in the context of the Centro Universitario de Investigación, Innovación y Diagnóstico Arterial (CUiiDARTE) project (Bia et al., 2011; Santana et al., 2012a,b), a population-based study developed in Uruguay. This includes data derived from community-based studies on demographic and anthropometric variables, exposure to CRFs, and personal and family history of cardiovascular disease (CVD), and data on hemodynamic, structural, and functional vascular parameters. Previous studies within the framework of the CUiiDARTE project allowed obtaining reference intervals, mean values, and standard deviation equations for regional arterial stiffness and stiffness gradients (Bia and Zócalo, 2021), macro- and micro-vascular reactivity (Zócalo and Bia, 2021a), global hemodynamics (Zócalo et al., 2020a), arterial blood flow velocities and Doppler-derived indexes (Zócalo and Bia, 2021b), and central BP waveform-derived indexes (Zócalo and Bia, 2021c).

All procedures were conducted in agreement with the Declaration of Helsinki. The protocol of the study was reviewed and approved by the Ethics Committee of Centro Hospitalario Pereira Rossell and Hospital de Clínicas, Universidad de la República. The participants provided their written informed consent to participate in this study. For adults, written informed consent was obtained prior to the evaluation. In subjects <18 y, parents' written consent and children's assent were obtained before the evaluations. Subjects or parents (in case of subjects aged <18 y) also provided informed written consent to have data from their medical records used for the purpose of this study.

### Anthropometric, Clinical, and Physical Activity Evaluation

Before cardiovascular assessment, a clinical interview, anthropometric evaluation, and blood tests (mainly in adults) were performed to assess for CRF exposure. Body weight (BW) and body height (BH) were measured with the participants wearing light clothing and no shoes. Standing BH was measured using a portable stadiometer and recorded to the nearest 0.1 cm. BW was measured with an electronic scale (841/843, Seca Inc., Hamburg, Germany; model HBF-514C, Omron Inc., Chicago, Illinois, United States) and recorded to the nearest 0.1 kg. Body mass index (BMI) was calculated as BW-to-squared BH ratio. In children and adolescents, *z*-scores for BMI (z-BMI) were calculated using the WHO software (Anthro-v.3.2.2; Anthro-Plus-v.1.0.4) (Castro et al., 2019). Obesity was defined as BMI ≥ 30 kg/m^2^ (subjects ≥18 y) or *z*-BMI ≥ 2 (subjects <18 y). History of dyslipidemia and diabetes were considered present if they had been previously diagnosed by referring physicians. In turn, dyslipidemia and diabetes diagnoses were based on plasma lipids and glucose levels (fasting plasma glucose ≥126 mg/dl, total cholesterol ≥240 mg/dl, or HDL cholesterol <40 mg/dl), respectively. Hypertension was considered present if it had been previously diagnosed; alternatively, high BP levels (hypertensive levels) were defined as brachial systolic BP (baSBP) ≥140 mmHg or brachial diastolic BP (baDBP) ≥90 mmHg (subjects ≥18 y), or as baSBP and baDBP >95th percentile for sex, age, and BH (subjects <18 y). Use of BP-, lipid- or glucose-lowering drugs was investigated. Regular or current smokers, defined as smoking at least one cigarette/week, were identified. Family history of CVD was defined by the presence of first-degree (for all the subjects) and/or second-degree (subjects ≤ 18 y) relatives with early (<55 y in males, <65 y in females) CVD. History of CVD was defined as the presence of valvular heart disease, cerebrovascular, coronary artery or peripheral arterial disease, impaired left ventricular ejection fraction, or hypertrophy. Cutoff values used to define the presence of CRFs were chosen, whenever possible, in accordance with clinical guidelines to enable optimal comparisons with data derived from other groups (Engelen et al., 2013, 2015; Bossuyt et al., 2015).

PA was determined using questionnaires and tested in several age groups for validity and reliability to measure its intensity, frequency, and duration during the week before the interview (Kowalski et al., 1997; Craig et al., 2003; Hurtig-Wennlöf et al., 2010; Bingham et al., 2016; Amor-Barbosa et al., 2021). In young children, specially developed and validated forms (questionnaires), involving interviewing parents, were considered (Bingham et al., 2016). The subjects were categorized dichotomously as (i) compliant (*n* = 1,969) or (ii) non-compliant (*n* = 1,650) with WHO-related PA recommendations. Accordingly, the subjects were considered compliant with international recommendations if they performed: (i) children (aged 3–4 y): ≥180 min of a variety of types of PA at any intensity, of which ≥60 min is moderate- to vigorous-intensity PA, spread throughout the day (World Health Organization, 2019); (ii) children and adolescents (aged 5–17 y): ≥60 min/day (average) of moderate-to-vigorous intensity PA (mostly aerobic) across the week; (iii) adults (aged 18–64 y): ≥150–300 min of moderate-intensity aerobic PA, ≥75–150 min of vigorous-intensity aerobic PA, or an equivalent combination of moderate-intensity and vigorous-intensity PA throughout the week; (iv) older adults (aged ≥65 y): same as in the other adults and a varied multicomponent PA that emphasizes on functional balance and strength training at moderate or greater intensity ≥3 days per week (World Health Organization, 2020).

### Cardiovascular Evaluation

The Participants were asked to avoid exercise, tobacco, alcohol, caffeine, and food intake 4 h prior to evaluation. All measurements were performed in a temperature-controlled environment (21–23°C), with the subjects in supine position, and after resting for at least 10–15 min, which ensured steady hemodynamic conditions. Using a validated oscillometric device (HEM-433INT; Omron Healthcare Inc., Illinois, United States), heart rate (HR) and baSBP and baDBP levels were recorded in the supine position simultaneously and/or immediately before or after each non-invasive arterial recording. Then, brachial artery pulse pressure (baPP; baPP = baSBP–baDBP) and mean BP (baMBP, baMBP = baDBP + baPP/3) were calculated.

**Figure 1:**
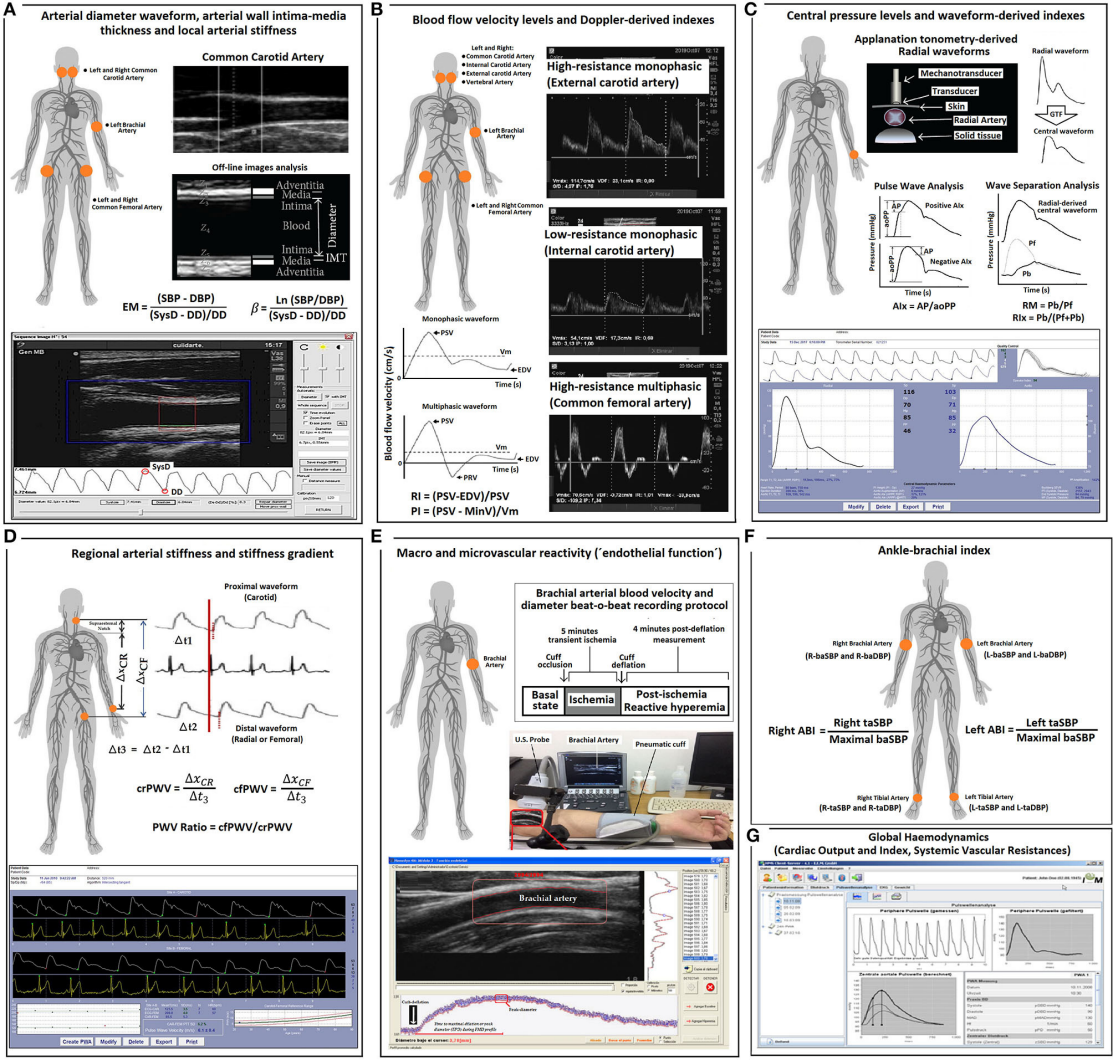
Summary of the methodology used for cardiovascular evaluations. **(A)** Arterial diameter, intima-media thickness and local arterial stiffness. EM, elastic modulus; SBP and DBP, systolic blood pressure and diastolic blood pressure, respectively. SysD and DD, systolic diameter and diastolic arterial diameter, respectively; IMT, intima-media thickness; **(B)** Blood flow velocity levels and Doppler-derived index. PSV, peak systolic velocity; Vm, mean velocity; EDV, end diastolic velocity; PRV, peak reversal (negative) velocity; RI and PI, Doppler-derived resistive index and pulsatile index; **(C)** Central (aortic) pressure and aortic waveform-derived indexes. GTF, general transfer function; AP, augmentation pressure; aoPP, aortic pulse pressure; AIx., augmentation index; Pf, forward pressure; Pb, backward pressure; RM, reflection magnitude; RIx, reflection index; **(D)** Regional arterial stiffness and stiffness gradient. crPWV and cfPWV, carotid-radial pulse wave velocity and carotid-femoral pulse wave velocity, respectively. **(E)** Macro- and micro-vascular reactivity. **(F)** Ankle brachial index (ABI). taSBP and baSBP, tibial artery systolic blood pressure and brachial artery systolic blood pressure, respectively. L-, left; R-, Right. **(G)** Global hemodynamic indexes derived by pulse contour analysis.

Figure 1 describes the non-invasive cardiovascular evaluation performed in the CUiiDARTE project.

#### Peripheral and Central Pressure and Aortic Wave-Derived Parameters

To assess central aortic BP (aoBP) levels and waveform-derived indexes, radial artery BP waveforms were recorded by applanation tonometry using Sphygmocor-CvMS (AtCor-Medical, Sidney Australia) (Figure 1) (Zinoveev et al., 2019; Mynard et al., 2020). Pressure signals were calibrated to baDBP and baMBP, and a generalized transfer-function was used to obtain corresponding aoBP waveforms and aortic systolic, diastolic, and pulse pressures (aoSBP, aoDBP, aoPP). Only adequate waveforms (visual inspection) and high-quality recordings (operator index ≥85) were considered. A step-by-step explanation of the methods used to obtain the aoBP can be found elsewhere (Zinoveev et al., 2019).

By means of pulse wave analysis (PWA), the first (P1) and second (P2) peaks of the aoBP wave were identified, and their height and time were determined. Then, the difference between P2 and P1 was computed as aortic augmented pressure (AP) and used to quantify aortic augmentation index (Aix = AP/aoPP). Since AIx depends on HR, AIx adjusted to a 75-beat/min HR (AIx@75) was calculated. The basic idea underlying PWA is that the forward wave travelling from the left ventricle toward the periphery is distally reflected, the reflected wave “augments” (central) pressure. AP represents the level of augmentation (a positive AP indicates “additional” pressure arising from reflections) (Baksi et al., 2009; Sugawara et al., 2010) and is calculated from the inflection point in the pressure wave (systolic phase) that “signalizes or identifies” the reflected component's arrival to the aortic root (Kelly et al., 1989). AIx is considered a surrogate index of wave reflection (although it is known that it also depends on factors like ventricle function) (Hametner and Wassertheurer, 2017).

Additionally, by means of wave separation analysis (WSA), forward and backward (Pf and Pb) components of the aortic pulse wave were quantified (Figure 1). While Pf represents the contribution of incident wave and re-reflections to recorded aoBP, Pb corresponds to the contribution of reflected wave. Finally, reflection magnitude (RM; RM = Pb/Pf) and reflection index (RIx; RIx = Pb/[Pf + Pb]) were obtained (Westerhof and Westerhof, 2013; Zamani et al., 2014). Importantly, whereas AIx, AIx@75, RM, and RIx can be determined without calibrating the pressure wave, AP, Pf, and Pb require calibration (Westerhof and Westerhof, 2013).

#### Brachial and Tibial Pressure and Ankle Brachial Index

Left and right baSBP and baDBP, and tibial artery systolic and diastolic BPs (taSBP, taDBP) were obtained at 5-min intervals (HEM-433INT; Omron Healthcare Inc., Lake Forest, IL, United States). At least five measurements were obtained from each recording site. Ankle brachial index (ABI), a marker of arterial permeability and central-peripheral BP amplification, was calculated (Zócalo and Bia, 2016):


$$\begin{array}{l} ABI=\frac{taSBP}{baSBP}. \end{array}$$

ABI values < 0.9 are conventionally used as a cutoff level to define peripheral obstructive arterial disease and increased cardiovascular risk.

#### Global Hemodynamic State

Systemic vascular resistance (SVR), cardiac output (CO), and cardiac index (CI; CI = CO/body surface area) were quantified by brachial artery pulse contour analysis (PCA) using Mobil-O-Graph (I.E.M.-GmbH, Stolberg, Germany) (Zócalo et al., 2020a,b). To this end, an oscillometric cuff was placed in the left arm. Cuff size was selected according to the arm size of the subjects. Only high-quality records (index ≤ 2) and satisfactory waves (visual inspection) were considered. Values of the subjects are the average of at least six consecutive recordings.

#### Arterial Diameter and Intima-Media Thickness

The left and right common carotid arteries (CCAs), common femoral artery (CFA), and left brachial artery (BA) were analyzed by ultrasound using M-Turbo (6–13 MHz: Sonosite Inc., Bothell, WA, United States) (Marin et al., 2020) (Figure 1). Sequences of images (30 s, B-Mode, longitudinal views) were stored for offline analysis. Beat-to-beat diameter waveforms were obtained using border detection software (Hemodyn-4M; Dinap s.r.l., Buenos Aires, Argentina). Peak systolic diameter (SysD), end-diastolic diameter (DD), and intima-media thickness (IMT, far wall, end diastole) values were obtained by averaging at least 20 beats. CCA diameter and IMT were measured a centimeter proximal to the carotid bulb, whereas CFA diameter and IMT were measured in a straight segment of the penultimate centimeter proximal to the bifurcation. BA measurements were performed at the elbow level in a straight segment of at least 1 cm long (Marin et al., 2020) (Figure 1).

#### Local Arterial Stiffness

Local arterial stiffness was quantified by means of elastic modulus (EM) and beta index (β). EM relates BP and arterial diameter changes (within a beat): EM = (SBP–DBP)/([SysD–DD]/DD) and measures the ability of the arteries to change its dimensions in response to the pulse pressure caused by cardiac ejection (pressure change required for [theoretic] 100% increase in diameter).

To minimize the impact that BP levels have on arterial stiffness, β was quantified: β = Ln(SBP/DBP)/([SysD–DD]/DD), where Ln is a natural logarithm. While oscillometry-derived baSBP and baDBP were used to quantify CFA and BA EMs and βs, aoSBP and aoDBP were used to quantify CCA EM and β (Figure 1).

#### Regional Arterial Stiffness and Stiffness Gradient

Carotid-femoral pulse wave velocity (cfPWV, a surrogate of aortic regional stiffness) and carotid-radial pulse wave velocity (crPWV, a surrogate of upper arm arteries stiffness) were obtained by applanation tonometry using Sphygmocor-CvMS (AtCor-Medical, Sidney Australia) (Bia and Zócalo, 2021) (Figure 1). We used direct distance multiplied by 0.8 for cfPWV (the “real cfPWV”), while subtracted distance was considered for crPWV quantification. cfPWV and crPWV values were obtained as the median of three measurements. PWV ratio (a marker of central-to-peripheral arterial stiffness gradients) was quantified as cfPWV/crPWV (Bia and Zócalo, 2021) (Figure 1).

Arterial stiffness (e.g., assessed by EM or PWV) is influenced by BP levels that were determined during the examination, which, if not considered, could lead to inaccurate conclusions (Bia and Zócalo, 2021). To overcome this issue, Shirai et al. (2006) proposed the use of cardio-ankle vascular index (CAVI). CAVI was suggested to better reflect structural changes in the arterial wall (with independence of arterial distending BP). In this study, CAVI cfPWV and CAVI crPWV were computed (Jurko et al., 2018) as follows:


$$\begin{array}{l} \text{CAVI}=\left(\text{Ln}\left[\text{baSBP}/\text{baDBP}\right]\right)*\left(\text{PWV}2*2\text{ρ}/\left[\text{baSBP}-\text{baDBP}\right]\right), \end{array}$$

where Ln is natural logarithm, and ρ is blood mass density (assumed to be 1,060 kg/m^3^). BP, cfPWV, or crPWV, and ρ were entered into the equation in Pa, m/s, and kg/m^3^, respectively.

#### Arterial Blood Flow Velocity and Doppler Indexes

Left and right CCA, internal carotid artery (ICA), external carotid artery (ECA), vertebral artery (VA), CFA, and left BA blood flow velocity waveforms were obtained by Doppler mode echography using M-Turbo (6–13 MHz; Sonosite Inc., Bothell, WA, United States) (Figure 1). The sample volume was placed 15–20 mm proximal or distal to CCA bifurcation, defined as the tip of the flow divider, to obtain (right and left) ICA, ECA, or CCA velocity waveforms (Oates et al., 2009). VA was identified between transverse processes of the vertebrae, and its Doppler data were obtained at the level of CCA bifurcation (V2 segment) (Kuhl et al., 2000). CFA data were obtained from the proximal straight arterial portion at the groin and BA from a straight segment of at least 1 cm (elbow level).

Peak systolic velocity (PSV), end-diastolic velocity (EDV), and mean blood flow velocity (Vm) levels were computed by drawing the blood flow velocity waveform envelope. Peak reversal diastolic velocity (PRV, reverse, backward, or upstream flow toward the central aorta) was also computed for the CFA (Figure 1). “Intra-beat” indexes, such as resistive index (RI) and pulsatility index (PI), were calculated (Figure 1) (Oates et al., 2009). These indexes are unit-less parameters independent of possible inaccuracies in the estimation of spectral velocity because of arterial diameter or angle correction (Oates et al., 2009). RI (RI = (PSV–EDV)/PSV) reflects the global resistance or arterial outflow needs of the downstream territory. RI is maximum (R = 1) in high-resistance territories (e.g., CFA) that lack end-diastolic blood flow (EDV = 0). PI reflects the vascular resistance of the downstream territory (even between territories of high resistance) and is calculated as: PI = (PSV–MinV)/Vm, where MinV is the minimal (negative or positive) velocity.

#### Macro- and Micro-Vascular Reactivity Indexes

Vascular reactivity (VR), defined as the capability of blood vessels to actively modify the diameter and flow resistance can be non-invasively assessed by analyzing vascular response to forearm occlusion. As in previous studies, VR was evaluated by means of standardized methods (Figure 1) (Zócalo and Bia, 2021a). The left shoulder and arm were positioned on a support, ensuring comfort and stability, thus avoiding muscle tension development and subsequent movement. Then, the left forearm and wrist were placed on a support to minimize motion and artifacts during recordings (e.g., due to cuff inflation). BA was examined on a longitudinal plane using M-Turbo (6–13 MHz: SonoSite Inc., Bothell, WA, United States). To ensure adequate recordings, the transducer was fixed using a stereotactic probe holder. Doppler and B-modes were selected to record BA center-line blood flow velocity and diameter, respectively. A standard pediatric BP cuff (Omron, Japan), positioned distally in the forearm, was inflated to 50 mmHg above baSBP for 5 min. Ultrasound-derived image sequences (videos) were obtained under the following conditions: (i) baseline (60-s videos obtained immediately before cuff inflation), (ii) occlusion [300-s videos recorded during the time the cuff remained inflated (distal transient ischemia)], and (iii) release [240-s videos recorded during cuff deflation and subsequent reactive hyperemia (RH)] (Zócalo and Bia, 2021a).

The acquired videos were stored for blind offline analysis with automatic wall detection and Doppler velocity tracing software (Hemodyn-4-M, Dinap s.r.l., Argentina; Sonosite Inc., United States). Once a straight segment of the BA was identified (B-mode recording) and the region of interest was established, the software allowed for the beat-to-beat automatic identification of arterial wall-lumen interfaces of the anterior and posterior walls, and instantaneous BA diameter was obtained (Figure 1). The following data were obtained under different conditions (e.g., baseline, RH): (i) BA SysD and DD, (ii) BA PSV and EDV, and (iii) BA RI, a measure of pulsate flow that reflects the resistance associated with distal microvessels. Values corresponding to RH state were at maximum levels observed within the first 210 s after cuff deflation. Pre-release arterial diameter and flow velocity were determined during the last 15–30 s before cuff deflation. Time to peak BA DD was calculated as the time from cuff deflation to maximum hyperemic BA DD.

With the data obtained during the VR test, flow-mediated dilation (FMD%) was calculated as follows (Celermajer et al., 1992):


$$\begin{array}{l} \text{FMD}\%\;=\frac{{\text{DD}}_{\text{Peak}}-{\text{DD}}_{\text{Basal}}}{{\text{DD}}_{\text{Basal}}}*100, \end{array}$$

where DD_Peak_ and DD_Basal_ are maximum BA DD (RH state) and baseline BA DD, respectively. Different FMD% temporal-patterns have been described, with differences in the kinetics of dilatory response (Irace et al., 2014, 2018; Zócalo et al., 2017). The magnitude and kinetics (e.g., latency) of vasodilatory response would give complimentary information (Irace et al., 2018). We quantified the time to peak diameter (TPD), reflecting the time to maximal BA DD or maximal dilation after cuff deflation (RH state) (TPD_FMD%).

Whereas, FMD% provides data on endothelial function “recruitability” (Celermajer et al., 1992), it does not provide information related with basal or tonic reactivity (e.g., basal release of vasoactive factors) (Gori et al., 2008, 2010; Torrado et al., 2015a,b). A low baseline tone, leading to pre-dilated BA, could result in a blunted FMD% despite normal endothelial function. In turn, high baseline tone, associated with pre-constricted BA, could result in normal FMD% despite abnormal endothelial function. Therefore, FMD% does not provide data on endothelial responsiveness to resting wall shear stress levels, or on vasoconstrictor response to shear stress reductions (Gori et al., 2010). To assess the arterial response to low blood flow (“vaso-constriction”), Gori et al. (2008) proposed an index that considers data obtained during cuff inflation (arterial occlusion). Similar to FMD%, the response observed under conditions of reduced blood flow was named low-flow mediated (vaso) constriction (LFMC). LFMC% could provide (complementary) data for characterization of vessel responsiveness and/or risk stratification. LFMC% is quantified as percentage change in BA DD, considering basal and pre-release data (Zócalo and Bia, 2021a):


$$\begin{array}{l} \text{LFMC}\%=\left(\text{DD\_prerelease}-\text{DD\_basal}\right)/\text{DD\_basal}*100. \end{array}$$

It was proposed that the combined evaluation of vasodilatory and vasoconstrictory responses (FMD% and LFMC%), as well as their composite endpoint, total vasoactive range, or vascular reactivity (TVR) may improve risk stratification (Gori et al., 2010). TVR was quantified as TVR = (DD_peak–DD_pre-release)/DD_basal ^*^100.

Finally, flow velocity and distal resistance changes (Doppler-derived) during RH have been proposed to evaluate distal microvessels reactivity (Zócalo and Bia, 2021a). Microvascular reactivity during RH was evaluated as ΔRI% = ([BA RI peak–BA RI basal]/BA RI basal)^*^100.

### Statistical Analysis

A stepwise analysis was performed. First, descriptive statistics were obtained for the 3,619 subjects (Table 1; Supplementary Table 1).

**Table 1:** Characteristics of the subjects according to compliance with physical activity recommendations (CPARs).

**Variable**	**Not-CPAR group (*****n*** **= 1,650)**				**CPAR group (*****n*** **= 1,969)**				**All subjects (*****n*** **= 3,619)**			
	**MV**	**SD**	**Min.**	**Max.**	**MV**	**SD**	**Min.**	**Max.**	**MV**	**SD**	**Min.**	**Max.**
Female sex (%)	48.90				41.20				44.70			
Age (years)	40.2	23.0	4.2	86.3	29.49*	24.52	3.9	88.8	34.39	24.44	3.9	88.8
Family CVD (%)	16.7				12.4*				14.4			
Body weight (Kg)	71.63	21.86	16.02	150.60	54.41*	26.07	13.2	134.7	62.24	25.72	13.2	150.6
Body height (m)	1.62	0.15	1.07	1.96	1.50*	0.26	0.97	1.97	1.55	0.23	0.97	1.97
BMI (Kg/m^2)^	26.66	6.13	11.53	71.34	22.37*	5.36	12.27	45.52	24.32	6.11	11.53	71.34
TC (mg/dl)	203.4	42.9	94.3	354	199.23	43.08	101	363	201.49	43.04	94.3	363
HDL (mg/dl)	50.3	14.2	17	109	54.30*	14.39	23	103	52.20	14.44	17	109
LDL (mg/dl)	126.1	38.7	31	254	122.19*	39	35	293	124.30	38.92	31	293
Triglicerides (mg/dl)	139.9	87.7	26	783	112.99*	68.01	24	504	127.15	80.12	24	783
Atherogenic Index	4.31	1.39	1.56	11	3.84*	1.16	1.14	11.6	4.09	1.31	1.14	11.6
Glicaemia (mg/dl)	96.8	20.9	47	296	93.95	17.24	59	307	95.45	19.26	47	307
Creatinine (mg/dl)	0.89	0.3	0.38	2.38	0.88	0.23	0.35	2.18	0.88	0.27	0.35	2.38
TC >240 mg/dl (%)	8.7				5.7*				7.1			
HDL. <40 mg/dl (%)	10.5				4.8*				7.4			
Glic. >126 mg/dl (%)	1.7				0.6*				1.1			
Current smoker (%)	14.5				6.3*				10.0			
Hypertension (%)	33.5				18.7*				25.5			
Diabetes (%)	8.3				2.7*				5.3			
History of CVD (%)	9.9				4.1*				6.8			
Anti-hypertens. (%)	28.5				17.0*				22.3			
Anti-hyperlip. (%)	20.3				12.9*				16.3			
Anti-diabetic (%)	7.0				2.4*				4.5			
Obesity (%)	33.0				16.7*				24.1			

*MV, mean value; SD, standard deviation; Min, minimal value; Max, maximal value; CPARs, compliance with physical activity recommendations; CVD, cardiovascular disease; Family CVD, family history of CVD; BMI, body mass index; TC, total cholesterol; HDL and LDL, HDL and LDL cholesterol; Anti-hypertens, anti-hypertension drugs; Anti-hyperlip., anti-hyperlipidemic drugs; Anti-diabetic, anti-diabetic drugs*.

* *p < 0.05. non-CPAR group vs. CPAR group*.

#### Compliance With Physical Activity Recommendations and Cardiovascular Risk Factors

Second, we evaluate the association between CPARs (1: yes, 0: no) and levels of different blood and anthropometric variables usually measured to assess the presence of CRFs: BW (in kg), BH (in m), BMI (in kg/m^2^), total, HDL, and LDL cholesterol levels (in mg/dl), triglycerides (in mg/dl), atherogenic index (total/HDL cholesterol ratio), glycemia (in mg/dl), and creatinine (in mg/dl) (Table 2). To this end, two-tailed partial point bi-serial correlations were performed (cofactors: age, sex, on antihyperlipidemic and antidiabetic therapy). An association was considered significant if the *p*-value was < 0.05 and the 95% confidence interval of Pearson's coefficient, quantified by Bootstrapping, did not contain a 0 value. Bootstrap-derived 95% confidence interval limits (1,000 samples) were determined by applying bias-corrected and accelerated methods.

**Table 2:** Partial correlations between CPARs and cardiovascular risk factors.

	**R**	***p***	**Bootstrap (1,000 samples)**	
			**95% C.I., L.L.**	**95% C.I., U.L.**
Body weight (Kg)	−0.228	<0.001	−0.318	−0.134
Body height (m)	0.051	0.280	−0.041	0.147
BMI (Kg/m^2^)	−0.295	<0.001	−0.376	−0.207
Total cholesterol (mg/dl)	−0.055	0.237	−0.148	0.032
HDL cholesterol (mg/dl)	0.178	<0.001	0.091	0.260
LDL cholesterol (mg/dl)	−0.049	0.291	−0.143	0.043
Triglicerides (mg/dl)	−0.231	<0.001	−0.319	−0.136
Atherogenic Index	−0.214	<0.001	−0.298	−0.127
Glicaemia (mg/dl)	−0.121	0.009	−0.198	−0.042
Creatinine (mg/dl)	−0.109	0.019	−0.198	−0.007

*BMI, body mass index; Cofactors, age, sex, antihyperlipidemic agent, antidiabetic agents; Bootstrap, bootstraping (1,000 samples); CI, confidence interval; CI: type, bias-corrected and accelerated (BCa); r, correlation coefficient; L.L., lower limit; U.L., upper limit; CPARs (1: yes, 0: no)*.

After showing that CPARs is associated with most of the variables underlying CRFs, we suggest that the remaining evaluations would include adjustments of all the quantified CRFs.

#### Compliance With Physical Activity Recommendations and the Cardiovascular System

Third, we evaluated the association between CPARs and cardiovascular properties using multiple linear regression models that included interaction analysis (CPAR^*^age) (Tables 3–11; Supplementary Tables 2–95). The variables “y,” “x,” and “w” (moderating variables) were assigned, respectively, to the cardiovascular variable (y), CPARs (x), and age (w). In all cases, the following covariates (1: yes or female, 0: no or male) were entered in the models (“enter” method): age (y), sex, total cholesterol >240 mg/dl, HDL cholesterol <40 mg/dl, glycemia >126 mg/dl, current smoker, hypertension, diabetes, CVD, antihypertensive agents, antihyperlipidemic agent, antidiabetic agents, and obesity.

**Table 3:** Independent association between CPARs and global hemodynamic indexes, with age as a moderating variable.

**Independent variable**	**β**	***p***	**R**	**R^**2**^**	***p***
**Cardiac Output (y)**					
Constant	5.5254	<0.001	0.382	0.146	<0.001
CPAR (1: yes, 0: no)	−0.683	<0.001			
Age (years)	−0.006	<0.001			
CPAR*Age	0.0127	<0.001			
Sex (1: female, 0: male)	−0.337	<0.001			
Total cholesterol > 240 mg/dl	−0.233	0.006			
Hypertension (1: yes, 0: no)	0.1648	0.016			
Antihyperlipidemic agent (1: yes, 0: no)	−0.202	0.012			
Obesity (1: yes, 0: no)	0.1409	0.004			
**Cardiac Index (y)**					
Constant	3.7912	<0.001	0.417	0.173	<0.001
CPAR (1: yes, 0: no)	0.8678	<0.001			
Age (years)	−0.017	<0.001			
CPAR*Age	−0.019	<0.001			
**Systemic Vascular Resistances (y)**					
Constant	0.956	<0.001	0.51	0.261	<0.001
CPAR (1: yes, 0: no)	0.084	<0.001			
Age (years)	0.004	<0.001			
CPAR*Age	−0.002	<0.001			
Sex (1: female, 0: male)	0.053	<0.001			
Total cholesterol > 240 mg/dl	0.061	0.002			
Hypertension (1: yes, 0: no)	0.040	0.011			
Obesity (1: yes, 0: no)	−0.025	0.030			
**Heart Rate (y)**					
Constant	78.234	<0.001	0.549	0.301	<0.001
CPAR (1: yes, 0: no)	5.290	<0.001			
Age (years)	−0.279	<0.001			
CPAR*Age	−0.136	<0.001			
Sex (1: female, 0: male)	4.021	<0.001			
Current smoke (1: yes, 0: no)	−5.261	<0.001			
Antihypertensive agents (1: yes, 0: no)	3.900	0.012			
Obesity (1: yes, 0: no)	2.305	0.008			
**Brachial Artery Mean Blood Pressure (y)**					
Constant	80.135	<0.001	0.643	0.414	<0.001
CPAR (1: yes, 0: no)	−4.646	<0.001			
Age (years)	0.239	<0.001			
CPAR*Age	0.049	0.030			
Sex (1: female, 0: male)	−1.594	0.002			
Hypertension (1: yes, 0: no)	5.399	<0.001			
Diabetes (1: yes, 0: no)	3.679	0.037			
CVD (1: yes, 0: no)	−4.471	0.003			
Antihypertensive agents (1: yes, 0: no)	−2.575	0.020			

*Y, dependent variable; CPARs, compliance with physical activity recommendations; In all cases, CPARs (1: yes, 0: no) was included in the models as variable “X” and age as moderating variable “W”*.

**Table 4:** Association between CPARs and ankle brachial index, with age as a moderating variable.

**Independent variable**	**β**	***p***	**R**	**R^**2**^**	***p***	**β**	***p***	**R**	**R^**2**^**	***P***
	**Right baSBP (y)**					**Left baSBP (y)**				
Constant	110.821	<0.001	0.63	0.39	<0.0001	110.509	<0.001	0.63	0.39	<0.001
CPAR (1: yes, 0: no)	−6.079	<0.001				−5.914	<0.001			
Age (years)	0.298	<0.001				0.297	<0.001			
CPAR*Age	0.089	<0.001				0.087	<0.001			
Sex (1: female, 0: male)	−3.747	<0.001				−3.007	<0.001			
Hypertension (1: yes, 0: no)	8.428	<0.001				8.695	<0.001			
Diabetes (1: yes, 0: no)	5.045	0.001				5.004	0.001			
CVD (1: yes, 0: no)	−6.332	<0.001				−4.549	<0.001			
Antihypertensive agents (1: yes, 0: no)	−3.090	0.003				−3.260	0.001			
Antihyperlipidemic agent (1: yes, 0: no)	−2.153	0.012				−2.645	0.002			
Antidiabetic agents (1: yes, 0: no)	–	–				−3.511	0.028		
Obesity (1: yes, 0: no)	2.286	<0.001				2.867	<0.001			
	**Right baDBP (y)**					**Left baDBP (y)**				
Constant	61.300	<0.001	0.62	0.38	<0.0001	63.108	<0.001	0.543	0.29	<0.001
CPAR (1: yes, 0: no)	−2.721	<0.001				−2.783	<0.001			
Age (years)	0.232	<0.001				0.187	<0.001			
CPAR*Age	0.034	0.027				0.035	0.022			
Sex (1: female, 0: male)	−1.060	0.005				−1.087	0.003			
Total cholesterol > 240 mg/dl	2.814	<0.001				2.175	0.001			
Hypertension (1: yes, 0: no)	3.373	<0.001				3.071	<0.001			
Diabetes (1: yes, 0: no)	2.528	0.017				2.367	0.026			
CVD (1: yes, 0: no)	−3.631	<0.001				−2.915	<0.001			
Antihypertensive agents (1: yes, 0: no)	−1.595	0.025				–	–			
Obesity (1: yes, 0: no)	1.790	<0.001				1.991	<0.001			
	**Right taSBP (y)**					**Left taSBP (y)**				
Constant	124.499	<0.001	0.63	0.39	<0.0001	124.428	<0.001	0.64	0.41	<0.001
CPAR (1: yes, 0: no)	−5.389	<0.001				−4.967	<0.001			
Age (years)	0.433	<0.001				0.451	<0.001			
CPAR*Age	0.077	0.006				0.055	0.045			
Sex (1: female, 0: male)	−7.507	<0.001				−7.401	<0.001			
Current smoke (1: yes, 0: no)	−2.742	0.016				−2.346	0.034			
Hypertension (1: yes, 0: no)	7.885	<0.001				8.486	0.000			
Diabetes (1: yes, 0: no)	3.997	0.042				4.088	0.035			
CVD (1: yes, 0: no)	−11.171	<0.001				−10.060	<0.001			
Antihypertensive agents (1: yes, 0: no)	−2.895	0.028				−3.127	0.016			
Antihyperlipidemic agent (1: yes, 0: no)	−2.860	0.008				−3.141	0.003			
Antidiabetic agents (1: yes, 0: no)	–	–				−4.317	0.036			
Obesity (1: yes, 0: no)	2.686	0.001				2.813	0.001			
	**Right taDBP (y)**					**Left taDBP (y)**				
Constant	63.234	<0.001	0.51	0.26	<0.0001	63.995	<0.001	0.53	0.28	<0.001
CPAR (1: yes, 0: no)	−2.751	<0.001				−2.910	<0.001			
Age (years)	0.183	<0.001				0.183	<0.001			
CPAR*Age	0.029	0.075				0.030	0.053			
Sex (1: female, 0: male)	−1.285	0.001				−1.381	<0.001			
Total cholesterol > 240 mg/dl	2.607	<0.001				2.183	0.002			
Hypertension (1: yes, 0: no)	2.981	<0.001				2.815	<0.001			
Diabetes (1: yes, 0: no)	3.004	0.008				3.101	0.005			
CVD (1: yes, 0: no)	−3.160	<0.001				−2.201	0.010			
Antihypertensive agents (1: yes, 0: no)	−1.581	0.038				–	–			
Antidiabetic agents (1: yes, 0: no)	−2.500	0.039				–	–			
Obesity (1: yes, 0: no)	1.379	0.004				1.487	0.001			
	**Right ABI (y)**					**Left ABI (y)**				
Constant	1.124	<0.001	0.25	0.06	<0.0001	1.128	<0.001	0.27	0.07	<0.001
CPAR (1: yes, 0: no)	0.013	0.037				0.019	0.003			
Age (years)	0.001	<0.001				0.001	<0.001			
CPAR*Age	0.000	0.110				0.000	0.001			
Sex (1: female, 0: male)	−0.025	<0.001				−0.028	<0.001			
HDL cholesterol <40 mg/dl	−0.012	0.043				–	–			
Current smoke (1: yes, 0: no)	−0.021	<0.001				−0.017	0.002			
Hypertension (1: yes, 0: no)	−0.013	0.016				−0.014	0.010			
Diabetes (1: yes, 0: no)	–	–				−0.019	0.045			
CVD (1: yes, 0: no)	−0.029	<0.001				−0.034	<0.001			

*Y, dependent variable; CPARs, compliance with physical activity recommendations. In all cases, CPARs (1: yes, 0: no) was included in the models as variable “X” and age as moderating variable “W”*.

**Table 5:** Association between CPARs and aortic pressure levels and waveform-derived indexes, with age as a moderating variable.

**Independent variable**	**B**	***p***	**R**	**R^**2**^**	***p***	**B**	***p***	**R**	**R^**2**^**	***p***
	**aoSBP (y)**					**aoDBP (y)**				
Constant	93.214	<0.001	0.652	0.425	<0.0001	62.245	<0.001	0.546	0.298	<0.001
CPAR (1: yes, 0: no)	−3.518	<0.001				−1.335	**0.076**			
Age (years)	0.344	<0.001				0.215	<0.001			
CPAR*Age	0.051	0.021				0.007	0.703			
Sex (1: female, 0: male)	−1.316	0.013				−1.000	0.014			
Total cholesterol > 240 mg/dl	–	–				2.198	0.004			
Hypertension (1: yes, 0: no)	5.764	<0.001				3.086	<0.001			
Diabetes (1: yes, 0: no)	4.110	0.007				–	–			
CVD (1: yes, 0: no)	−2.848	0.017				−4.516	<0.001			
Obesity (1: yes, 0: no)	1.389	0.030				1.701	0.001			
	**AP (y)**					**AIx (y)**				
Constant	−4.256	<0.001	0.735	0.54	<0.0001	−8.186	<0.001	0.716	0.513	<0.001
CPAR (1: yes, 0: no)	1.785	<0.001				4.982	<0.001			
Age (years)	0.211	<0.001				0.492	<0.001			
CPAR*Age	−0.035	<0.001				−0.098	<0.001			
Sex (1: female, 0: male)	1.786	<0.001				4.483	<0.001			
Total cholesterol > 240 mg/dl	–	–				2.775	0.002			
Hypertension (1: yes, 0: no)	1.136	0.001				–	–			
CVD (1: yes, 0: no)	–	–				−2.323	0.032			
	**AIx@75 (y)**					**Pf (y)**				
Constant	−8.031	<0.001	0.619	0.384	<0.0001	33.117	<0.001	0.204	0.042	<0.001
CPAR (1: yes, 0: no)	7.065	<0.001				−3.663	<0.001			
Age (years)	0.406	<0.001				−0.063	<0.001			
CPAR*Age	−0.153	<0.001				0.072	<0.001			
Sex (1: female, 0: male)	5.697	<0.001				−1.665	<0.001			
Total cholesterol > 240 mg/dl	2.578	0.007				−2.044	0.005			
Current smoke (1: yes, 0: no)	–	–				1.269	0.037			
Hypertension (1: yes, 0: no)	1.838	0.023				2.119	0.001			
Diabetes (1: yes, 0: no)	–	–				2.506	0.028			
CVD (1: yes, 0: no)	−2.482	0.032				–	–			
	**Pb (y)**					**RM (Pb/Pf) (y)**				
Constant	10.532	<0.001	0.575	0.33	<0.0001	0.320	<0.001	0.66	0.436	<0.001
CPAR (1: yes, 0: no)	0.284	0.463				0.064	<0.001			
Age (years)	0.125	<0.001				0.005	<0.001			
CPAR*Age	−0.004	0.675				−0.001	<0.001			
Sex (1: female, 0: male)	–	–				0.048	<0.001			
Glicaemia> 126 mg/dl	2.189	0.021				–	–			
Total cholesterol > 240 mg/dl	–	–				0.030	0.007			
Hypertension (1: yes, 0: no)	1.451	<0.001				–	–			
Antihypertensive agents (1: yes, 0: no)	–	–				0.026	0.021			
Diabetes (1: yes, 0: no)	1.568	0.009				–	–			

*Y, dependent variable; CPARs, compliance with physical activity recommendations. In all cases, CPARs (1: yes, 0: no) was included in the models as variable “X” and age as moderating variable “W”*.

**Table 6:** Association between CPARs and regional arterial stiffness and stiffness gradient, with age as a moderating variable.

**Independent variable**	**B**	***p***	**R**	**R^**2**^**	***p***	**B**	***p***	**R**	**R^**2**^**	***p***
	**cfPWV (y)**					**CAVI cfPWV (y)**				
Constant	4.411	<0.001	0.84	0.70	<0.0001	2.811	<0.001	0.76	0.58	<0.0001
CPAR (1: yes, 0: no)	−0.051	0.631				0.271	0.414			
Age (years)	0.080	<0.001				0.190	<0.001			
CPAR*Age	0.000	0.983				−0.003	0.706			
Sex (1: female, 0: male)	−0.180	0.002				–	–			
Total cholesterol > 240 mg/dl	–	–				−0.666	0.045			
Current smoke (1: yes, 0: no)	–	–				−0.682	0.016			
Hypertension (1: yes, 0: no)	0.456	<0.001				0.911	0.001			
Diabetes (1: yes, 0: no)	1.580	<0.001				4.852	<0.001			
CVD (1: yes, 0: no)	−0.657	<0.001				−0.909	0.029			
Antihyperlipidemic agent (1: yes, 0: no)	−0.238	0.009				−0.650	0.024			
Obesity (1: yes, 0: no)	−0.224	0.001				−0.939	<0.001			
	**crPWV (y)**					**CAVI crPWV (y)**				
Constant	7.763	<0.001	0.64	0.41	<0.0001	12.070	<0.001	0.55	0.30	<0.0001
CPAR (1: yes, 0: no)	−0.002	0.987				0.146	0.740			
Age (years)	0.053	<0.001				0.126	<0.001			
CPAR*Age	−0.003	0.317				−0.010	0.333			
Sex (1: female, 0: male)	−0.535	<0.001				−1.608	<0.001			
Hypertension (1: yes, 0: no)	–	–				−1.332	<0.001			
Diabetes (1: yes, 0: no)	0.722	0.003				1.657	0.026			
CVD (1: yes, 0: no)	−0.623	0.001				−1.369	0.024			
Obesity (1: yes, 0: no)	−0.282	0.003				−1.061	<0.001			
	**PWV ratio (y)**					**CAVI PWV ratio (y)**				
Constant	0.598	<0.001	0.62	0.39	<0.0001	0.329	<0.001	0.60	0.36	<0.0001
CPAR (1: yes, 0: no)	−0.011	0.438				−0.022	0.400			
Age (years)	0.004	<0.001				0.008	<0.001			
CPAR*Age	0.001	0.134				0.001	0.108			
Sex (1: female, 0: male)	0.023	0.004				0.045	0.003			
Total cholesterol > 240 mg/dl	−0.032	0.040				−0.061	0.033			
Current smoke (1: yes, 0: no)	–	–				−0.047	0.046			
Hypertension (1: yes, 0: no)	0.064	<0.001				0.118	<0.001			
Diabetes (1: yes, 0: no)	0.074	0.002				0.157	<0.001			

*Y, dependent variable; CPARs, compliance with physical activity recommendations. In all cases, CPARs (1: yes, 0: no) was included in the models as variable “X” and age as moderating variable “W”*.

**Table 7:** Association between CPARs and local arterial stiffness, with age as a moderating variable.

**Independent variable**	**B**	***p***	**R**	**R^**2**^**	***p***	**B**	***p***	**R**	**R^**2**^**	***p***
	**Right CCA EM (y)**					**Left CCA EM (y)**				
Constant	230.59	<0.001	0.67	0.45	<0.0001	225.87	<0.001	0.742	0.551	<0.0001
CPAR (1: yes, 0: no)	36.83	0.247				20.12	0.461			
Age (years)	14.32	<0.001				15.18	<0.001			
CPAR*Age	−2.21	0.002				−1.28	0.036			
Current smoke (1: yes, 0: no)	−61.74	0.015				−72.15	0.002			
Hypertension (1: yes, 0: no)	146.25	<0.001				130.32	<0.001			
Diabetes (1: yes, 0: no)	131.23	0.006				–	–			
Antidiabetic agents (1: yes, 0: no)	−146.62	0.004				–	–			
Obesity (1: yes, 0: no)	92.56	<0.001				67.67	<0.001			
	**Right CCA beta index (y)**					**Left CCA beta index (y)**				
Constant	3.166	<0.001	0.64	0.41	<0.0001	3.110	<0.001	0.719	0.517	<0.0001
CPAR (1: yes, 0: no)	0.528	0.087				0.306	0.244			
Age (years)	0.130	<0.001				0.139	<0.001			
CPAR*Age	−0.023	0.001				−0.013	0.026			
Current smoke (1: yes, 0: no)	–	–				−0.574	0.010			
Hypertension (1: yes, 0: no)	0.932	<0.001				0.778	<0.001			
CVD (1: yes, 0: no)	–	–				1.289	<0.001			
Diabetes (1: yes, 0: no)	0.733	0.112				–	–			
Antidiabetic agents (1: yes, 0: no)	−1.157	0.018				–	–			
Obesity (1: yes, 0: no)	0.686	<0.001				0.426	0.011			
	**Right CFA EM (y)**					**Left CFA EM (y)**				
Constant	780.50	<0.001	0.33	0.11	<0.0001	832.59	<0.001	0.419	0.176	<0.0001
CPAR (1: yes, 0: no)	42.99	0.460				−73.66	0.203			
Age (years)	8.31	<0.001				10.03	<0.001			
CPAR*Age	−1.30	0.310				1.146	0.375			
Sex (1: female, 0: male)	−74.42	0.018				−124.59	<0.001			
Hypertension (1: yes, 0: no)	115.76	0.015				162.77	0.001			
Obesity (1: yes, 0: no)	124.30	0.001				–	–			
	**Right CFA beta index (y)**					**Left CFA beta index (y)**				
Constant	9.475	<0.001	0.23	0.05	<0.0001	9.994	<0.001	0.34	0.11	<0.0001
CPAR (1: yes, 0: no)	0.857	0.142				−0.388	0.505			
Age (years)	0.058	<0.001				0.076	<0.001			
CPAR*Age	−0.020	0.122				0.006	0.650			
Sex (1: female, 0: male)	–	–				−1.013	0.002			
CVD (1: yes, 0: no)	–	–				1.981	0.007			
Obesity (1: yes, 0: no)	0.888	0.016				–	–			
	**Left BA EM (y)**					**Left BA beta index (y)**				
Constant	1135.39	<0.001	0.39	0.15	<0.0001	12.933	<0.001	0.34	0.12	<0.0001
CPAR (1: yes, 0: no)	−356.03	0.004				−3.405	0.004			
Age (years)	7.656	<0.001				0.058	0.003			
CPAR*Age	4.373	0.064				0.041	0.070			
Sex (1: female, 0: male)	−267.80	<0.001				−2.340	<0.001			
Glicaemia> 126 mg/dl	−470.21	0.023				−3.950	0.046			
Antidiabetic agents (1: yes, 0: no)	353.70	0.018				3.646	0.011			
Obesity (1: yes, 0: no)	275.54	<0.001				2.366	0.000			

*Y, dependent variable; CPARs, compliance with physical activity recommendations. In all cases, CPARs (1: yes, 0: no) was included in the models as variable “X” and age as moderating variable “W”*.

**Table 8:** Association between CPARs and vascular reactivity indexes, with age as a moderating variable.

**Independent variable**	**B**	***p***	**R**	**R^**2**^**	***p***	**B**	***p***	**R**	**R^**2**^**	***p***
	**FMD% (y)**					**Time to maximal FMD% (y)**				
Constant	8.417	<0.001	0.281	0.079	<0.0001	93.573	<0.001	0.128	0.017	<0.0001
CPAR (1: yes, 0: no)	0.661	0.412				−7.850	0.320			
Age (years)	−0.061	<0.001				–	–			
CPAR*Age	−0.010	0.476				0.145	0.307			
Sex (1: female, 0: male)	0.662	0.029				–	–			
Current smoke (1: yes, 0: no)	–	–				10.342	0.012			
CVD (1: yes, 0: no)	–	–				11.083	0.024			
Obesity (1: yes, 0: no)	−0.905	0.010				–	–			
	**LFMC% (y)**					**TVR% (y)**				
Constant	4.968	<0.001	0.187	0.035	<0.0001	3.684	<0.001	0.2	0.04	<0.0001
CPAR (1: yes, 0: no)	0.777	0.385				−0.372	0.616			
Age (years)	−0.038	0.002				−0.029	0.005			
CPAR*Age	−0.016	0.329				0.011	0.433			
Sex (1: female, 0: male)	–	–				1.238	<0.001			
CVD (1: yes, 0: no)	–	–				−0.971	0.047			
	**ΔRI% (y)**					–	–	–	–	–
Constant	−26.133	<0.001	0.139	0.019	<0.0001	–	–	–	–	–
CPAR (1: yes, 0: no)	−0.274	0.884				–	–	–	–	–
CPAR*Age	−0.004	0.906				–	–	–	–	–
Current smoke (1: yes, 0: no)	1.828	0.046				–	–	–	–	–

*Y, dependent variable; CPARs, compliance with physical activity recommendations. In all cases, CPARs (1: yes, 0: no) was included in the models as variable “X” and age as moderating variable “W”*.

**Table 9:** Association between CPARs and arterial structural parameters, with age as a moderating variable.

**Independent variable**	**B**	***p***	**R**	**R^**2**^**	***p***	**B**	***p***	**R**	**R^**2**^**	***p***
	**Right CCA DD (y)**					**Left CCA DD (y)**				
Constant	5.681	<0.001	0.73	0.54	<0.001	5.573	0.000	0.77	0.59	<0.001
CPAR (1: yes, 0: no)	0.064	0.230				−0.040	0.414			
Age (years)	0.026	<0.001				0.027	0.000			
CPAR*Age	−0.001	0.387				0.001	0.503			
Sex (1: female, 0: male)	−0.385	<0.001				−0.424	0.000			
Current smoke (1: yes, 0: no)	0.146	0.001				0.136	0.001			
Hypertension (1: yes, 0: no)	0.132	0.002				0.148	0.000			
Diabetes (1: yes, 0: no)	0.380	<0.001				0.217	0.005			
CVD (1: yes, 0: no)	0.126	0.039				0.271	0.000			
Antihyperlipidemic agent (1: yes, 0: no)	–	–				−0.091	0.029			
Antidiabetic agents (1: yes, 0: no)	−0.176	0.036				–	–			
Obesity (1: yes, 0: no)	0.201	<0.001				0.216	0.000			
	**Right CCA IMT (y)**					**Left CCA IMT (y)**				
Constant	0.358	<0.001	0.78	0.61	<0.001	0.374	<0.001	0.76	0.58	<0.001
CPAR (1: yes, 0: no)	0.020	0.082				0.006	0.608			
Age (years)	0.007	<0.001				0.007	<0.001			
CPAR*Age	−0.001	0.033				0.000	0.325			
Sex (1: female, 0: male)	−0.016	0.008				−0.028	<0.001			
Total cholesterol > 240 mg/dl	0.013	0.212				0.034	0.004			
Current smoke (1: yes, 0: no)	–	–				0.022	0.030			
Hypertension (1: yes, 0: no)	0.030	0.001				0.032	0.002			
Diabetes (1: yes, 0: no)	0.050	0.003				–	–			
CVD (1: yes, 0: no)	–	–				0.047	0.001			
Antidiabetic agents (1: yes, 0: no)	0.057	0.001				0.046	0.020			
Obesity (1: yes, 0: no)	0.024	0.001				–	–			
	**Ratio right CCA IMT/DD (y)**					**Ratio left CCA IMT/DD (y)**				
Constant	0.067	<0.001	0.658	0.433	<0.001	0.071	<0.001	0.651	0.424	<0.001
CPAR (1: yes, 0: no)	0.002	0.273				0.001	0.607			
Age (years)	0.001	<0.001				0.001	<0.001			
CPAR*Age	0.000	0.110				0.000	0.328			
Sex (1: female, 0: male)	0.003	<0.001				0.002	0.004			
Total cholesterol > 240 mg/dl	–	–				0.006	<0.001			
Antidiabetic agents (1: yes, 0: no)	0.010	<0.001				0.005	0.043			
	**Right CFA DD (y)**					**Left CFA DD (y)**				
Constant	5.941	<0.001	0.741	0.55	<0.001	6.022	<0.001	0.774	0.598	<0.001
CPAR (1: yes, 0: no)	0.039	0.718				−0.343	0.001			
Age (years)	0.051	<0.001				0.051	<0.001			
CPAR*Age	0.003	0.221				0.011	<0.001			
Sex (1: female, 0: male)	−1.214	<0.001				−1.285	<0.001			
Current smoke (1: yes, 0: no)	0.201	0.027				0.260	0.004			
Diabetes (1: yes, 0: no)	–	–				−0.360	0.032			
CVD (1: yes, 0: no)	−0.418	0.001				−0.486	<0.001			
Antihypertensive agents (1: yes, 0: no)	–	–				−0.239	0.025			
Obesity (1: yes, 0: no)	0.297	<0.001				0.259	<0.001			
	**Right CFA IMT (y)**					**Left CFA IMT (y)**				
Constant	0.310	<0.001	0.547	0.299	<0.001	0.338	0.001	0.469	0.22	<0.001
CPAR (1: yes, 0: no)	0.027	0.717				−0.081	0.501			
Age (years)	0.009	<0.001				0.011	<0.001			
CPAR*Age	−0.002	0.252				0.000	0.943			
	**Left BA DD (y)**					**Left BA IMT (y)**				
Constant	3.133	<0.001	0.764	0.584	<0.001	0.285	<0.001	0.593	0.352	<0.001
CPAR (1: yes, 0: no)	−0.306	<0.001				−0.003	0.879			
Age (years)	0.021	<0.001				0.002	<0.001			
CPAR*Age	0.005	<0.001				0.000	0.709			
Sex (1: female, 0: male)	−0.741	<0.001				−0.037	<0.001			
Current smoke (1: yes, 0: no)	0.206	<0.001				–	–			
Antidiabetic agents (1: yes, 0: no)	–	–				0.047	0.017			

*Y, dependent variable; CPARs, compliance with physical activity recommendations. In all cases, CPAR (1: yes, 0: no) was included in the models as variable “X” and age as moderating variable “W.”*

**Table 10:** Association between CPARs and arterial hemodynamic parameters, with age as a moderating variable.

**Independent variable**	**B**	***p***	**R**	**R^**2**^**	***p***	**B**	***p***	**R**	**R^**2**^**	***p***
	**Right CCA PSV (y)**					**Left CCA PSV (y)**				
Constant	128.62	<0.001	0.715	0.512	<0.0001	134.15	<0.001	0.711	0.506	<0.0001
CPAR (1: yes, 0: no)	0.307	0.843				0.887	0.568			
Age (years)	−0.782	<0.001				−0.811	<0.001			
CPAR*Age	0.011	0.740				0.027	0.443			
Sex (1: female, 0: male)	−8.535	<0.001				−8.939	<0.001			
Hypertension (1: yes, 0: no)	3.195	0.011				3.532	0.008			
CVD (1: yes, 0: no)	−3.834	0.030				−4.333	0.020			
Obesity (1: yes, 0: no)	−1.560	0.106				–	–			
	**Right CCA EDV (y)**					**Left CCA EDV (y)**				
Constant	31.97	<0.001	0.592	0.351	<0.0001	32.81	<0.001	0.591	0.35	<0.0001
CPAR (1: yes, 0: no)	0.149	0.748				0.484	0.296			
Age (years)	−0.164	<0.001				−0.166	<0.001			
CPAR*Age	−0.001	0.934				−0.008	0.460			
Sex (1: female, 0: male)	−0.716	0.004				–	–			
Total cholesterol > 240 mg/dl	1.107	0.013				1.244	0.008			
Current smoke (1: yes, 0: no)	–	–				1.062	0.007			
Diabetes (1: yes, 0: no)	–	–				−1.863	0.010			
	**Right ICA PSV (y)**					**Left ICA PSV (y)**				
Constant	99.87	<0.001	0.545	0.297	<0.0001	103.15	<0.001	0.59	0.348	<0.0001
CPAR (1: yes, 0: no)	−0.016	0.993				3.634	0.031			
Age (years)	−0.578	<0.001				−0.631	<0.001			
CPAR*Age	0.027	0.459				−0.043	0.236			
HDL cholesterol <40 mg/dl	–	–				−3.123	0.045			
Glicaemia> 126 mg/dl	10.621	0.006				9.655	0.015			
Current smoke (1: yes, 0: no)	−3.613	0.009				−3.388	0.017			
Hypertension (1: yes, 0: no)	3.712	0.006				2.930	0.038			
CVD (1: yes, 0: no)	4.640	0.014				–	–			
Obesity (1: yes, 0: no)	−2.231	0.031				–	–			
	**Right ICA EDV (y)**					**Left ICA EDV (y)**				
Constant	37.09	<0.001	0.549	0.302	<0.0001	38.05	<0.001	0.589	0.347	<0.0001
CPAR (1: yes, 0: no)	0.694	0.305				2.631	<0.001			
Age (years)	−0.212	<0.001				−0.231	<0.001			
CPAR*Age	−0.007	0.621				−0.041	0.005			
Sex (1: female, 0: male)	1.269	<0.001				1.238	0.001			
Total cholesterol > 240 mg/dl	1.299	0.033				2.396	<0.001			
Diabetes (1: yes, 0: no)	–	–				−2.522	0.013			
Glicaemia> 126 mg/dl	3.819	0.012				–	–			
	**Right ECA PSV (y)**					**Left ECA PSV (y)**				
Constant	105.48	<0.001	0.32	0.102	<0.0001	105.90	<0.001	0.332	0.11	<0.0001
CPAR (1: yes, 0: no)	−1.940	0.359				−2.904	0.131			
Age (years)	−0.236	<0.001				−0.302	<0.001			
CPAR*Age	0.046	0.306				0.051	0.223			
Sex (1: female, 0: male)	−13.157	<0.001				−11.479	<0.001			
Hypertension (1: yes, 0: no)	4.838	0.004				5.087	0.002			
CVD (1: yes, 0: no)	–	–				−6.015	0.007			
Antihyperlipidemic agent (1: yes, 0: no)	–	–				3.676	0.019			
	**Right ECA EDV (y)**					**Left ECA EDV (y)**				
Constant	14.468	<0.001	0.278	0.077	<0.0001	14.912	<0.001	0.258	0.066	<0.0001
CPAR (1: yes, 0: no)	−1.146	0.023				−0.759	0.114			
CPAR*Age	0.016	0.143				0.010	0.335			
Sex (1: female, 0: male)	−2.420	<0.001				−2.386	<0.001			
Glicaemia> 126 mg/dl	−2.582	0.022				0.969	0.035			
Current smoke (1: yes, 0: no)	2.431	<0.001				2.210	<0.001			
	**Right VA PSV (y)**					**Left VA PSV (y)**				
Constant	60.505	<0.001	0.312	0.097	<0.0001	68.187	<0.001	0.553	0.306	<0.0001
CPAR (1: yes, 0: no)	2.892	0.145				6.581	<0.001			
Age (years)	−0.245	<0.001				−0.367	<0.001			
CPAR*Age	−0.026	0.587				−0.106	0.003			
Current smoke (1: yes, 0: no)	–	–				−3.465	0.026			
Antihypertensive agents (1: yes, 0: no)	–	–				4.901	0.004			
Obesity (1: yes, 0: no)	–	–				−2.639	0.012			
	**Right VA EDV (y)**					**Left VA EDV (y)**				
Constant	16.396	<0.001	0.267	0.072	<0.0001	18.193	<0.001	0.379	0.144	<0.0001
CPAR (1: yes, 0: no)	0.066	0.899				1.835	<0.001			
Age (years)	−0.054	<0.001				−0.066	<0.001			
CPAR*Age	0.000	0.989				−0.034	0.007			
Sex (1: female, 0: male)	0.962	0.002				0.896	0.003			
Total cholesterol > 240 mg/dl	1.198	0.037				–	–			
Obesity (1: yes, 0: no)	–	–				−0.741	0.040			
	**Right CFA PSV (y)**					**Left CFA PSV (y)**				
Constant	135.454	<0.001	0.47	0.221	<0.0001	133.324	<0.001	0.427	0.183	<0.0001
CPAR (1: yes, 0: no)	−6.141	0.012				−5.615	0.019			
Age (years)	−0.611	<0.001				−0.558	<0.001			
CPAR*Age	0.061	0.240				0.071	0.172			
Sex (1: female, 0: male)	10.033	<0.001				9.416	<0.001			
Hypertension (1: yes, 0: no)	6.012	0.002				–	–			
Antidiabetic agents (1: yes, 0: no)	−10.120	0.007				–	–			
	**Right CFA PRV (y)**					**Left CFA PRV (y)**				
Constant	−31.143	<0.001	0.233	0.054	<0.0001	−30.802	<0.001	0.247	0.061	<0.0001
CPAR (1: yes, 0: no)	0.465	0.663				2.685	0.006			
Age (years)	−0.084	<0.001				−0.069	<0.001			
CPAR*Age	−0.006	0.804				−0.055	0.009			
Sex (1: female, 0: male)	3.764	<0.001				3.778	<0.001			
HDL cholesterol <40 mg/dl	2.076	0.027				–	–			
Hypertension (1: yes, 0: no)	2.203	0.009				–	–			
CVD (1: yes, 0: no)	3.342	0.006				–	–			
Obesity (1: yes, 0: no)	2.094	0.001				–	–			
	**Left BA PSV (y)**					**Left BA EDV (y)**				
Constant	84.310	<0.001	0.452	0.204	<0.0001	1.474	0.091	0.191	0.037	<0.0001
CPAR (1: yes, 0: no)	10.448	<0.001				4.694	<0.001			
Age (years)	−0.324	<0.001				0.007	0.694			
CPAR*Age	−0.170	<0.001				−0.077	<0.001			
Current smoke (1: yes, 0: no)	–	–				2.890	<0.001			
Hypertension (1: yes, 0: no)	3.718	0.023				–	–			
Diabetes (1: yes, 0: no)	6.782	0.018				–	–			
CVD (1: yes, 0: no)	−4.505	0.033				–	–			
Obesity (1: yes, 0: no)	–	–				1.162	0.039			

*Y, dependent variable; CPARs, compliance with physical activity recommendations. In all cases, CPARs (1: yes, 0: no) was included in the models as variable “X” and age as moderating variable “W.”*

**Table 11:** Association between CPARs and blood flow Doppler-derived indexes, with age as a moderating variable.

**Independent variable**	**B**	***p***	**R**	**R^**2**^**	***p***	**B**	***p***	**R**	**R^**2**^**	***p***
	**Right CCA RI (y)**					**Left CCA RI (y)**				
Constant	0.752	<0.001	0.257	0.066	<0.001	0.756	<0.001	0.283	0.08	<0.001
CPAR (1: yes, 0: no)	0.000	0.996				−0.001	0.820			
Age (years)	0.000	<0.001				−0.001	<0.001			
CPAR*Age	0.000	0.896				0.000	0.223			
Sex (1: female, 0: male)	−0.019	<0.001				−0.021	<0.001			
Total cholesterol > 240 mg/dl	−0.019	<0.001				−0.020	<0.001			
Current smoke (1: yes, 0: no)	−0.015	<0.001				−0.018	<0.001			
Diabetes (1: yes, 0: no)	–	–				0.016	0.028			
	**Right ICA RI (y)**					**Left ICA RI (y)**				
Constant	0.621	<0.001	0.171	0.029	<0.001	0.621	<0.001	0.185	0.034	<0.001
CPAR (1: yes, 0: no)	−0.005	0.376				−0.007	0.202			
CPAR*Age	0.000	0.214				0.000	0.198			
Sex (1: female, 0: male)	−0.007	0.027				−0.006	0.039			
Total cholesterol > 240 mg/dl	−0.016	0.004				−0.021	<0.001			
Current smoke (1: yes, 0: no)	−0.016	0.001				−0.019	<0.001			
Hypertension (1: yes, 0: no)	0.012	0.013				0.015	0.002			
	**Right ECA RI (y)**					**Left ECA RI (y)**				
Constant	0.862	<0.001	0.279	0.004	<0.001	0.858	<0.001	0.269	0.072	<0.001
CPAR (1: yes, 0: no)	0.008	0.130				0.001	0.886			
Age (years)	−0.001	<0.001				−0.001	<0.001			
CPAR*Age	0.000	0.481				0.000	0.849			
Sex (1: female, 0: male)	–	–				0.006	0.014			
Total cholesterol > 240 mg/dl	−0.013	0.007				–	–			
Glicaemia> 126 mg/dl	0.030	0.009				–	–			
Current smoke (1: yes, 0: no)	−0.031	<0.001				−0.024	<0.001			
Antihypertensive agents (1: yes, 0: no)	0.012	0.011				–	–			
Obesity (1: yes, 0: no)	−0.008	0.017				–	–			
	**Right VA RI (y)**					**Left VA RI (y)**				
Constant	0.724	<0.001	0.199	0.04	<0.001	0.726	<0.001	0.23	0.053	<0.001
CPAR (1: yes, 0: no)	0.013	0.126				−0.002	0.762			
Age (years)	0.000	0.008				−0.001	<0.001			
CPAR*Age	0.000	0.421				0.000	0.402			
Sex (1: female, 0: male)	−0.017	0.001				−0.015	0.001			
Current smoke (1: yes, 0: no)	–	–				−0.034	<0.001			
	**Right CFA PI (y)**					**Left CFA PI (y)**				
Constant	4.644	<0.001	0.282	0.079	<0.001	5.187	<0.001	0.213	0.045	<0.001
CPAR (1: yes, 0: no)	1.900	0.007				1.441	**0.092**			
Age (years)	0.105	<0.001				0.089	<0.001			
CPAR*Age	−0.022	0.146				−0.023	0.212			
Sex (1: female, 0: male)	−1.451	<0.001				−1.256	0.006			
Obesity (1: yes, 0: no)	–	–				−1.962	<0.001			

*Y, dependent variable; CPARs, compliance with physical activity recommendations. In all cases, CPARs (1: yes, 0: no) was included in the models as variable “X” and age as moderating variable “W”*.

**Figure 2:**
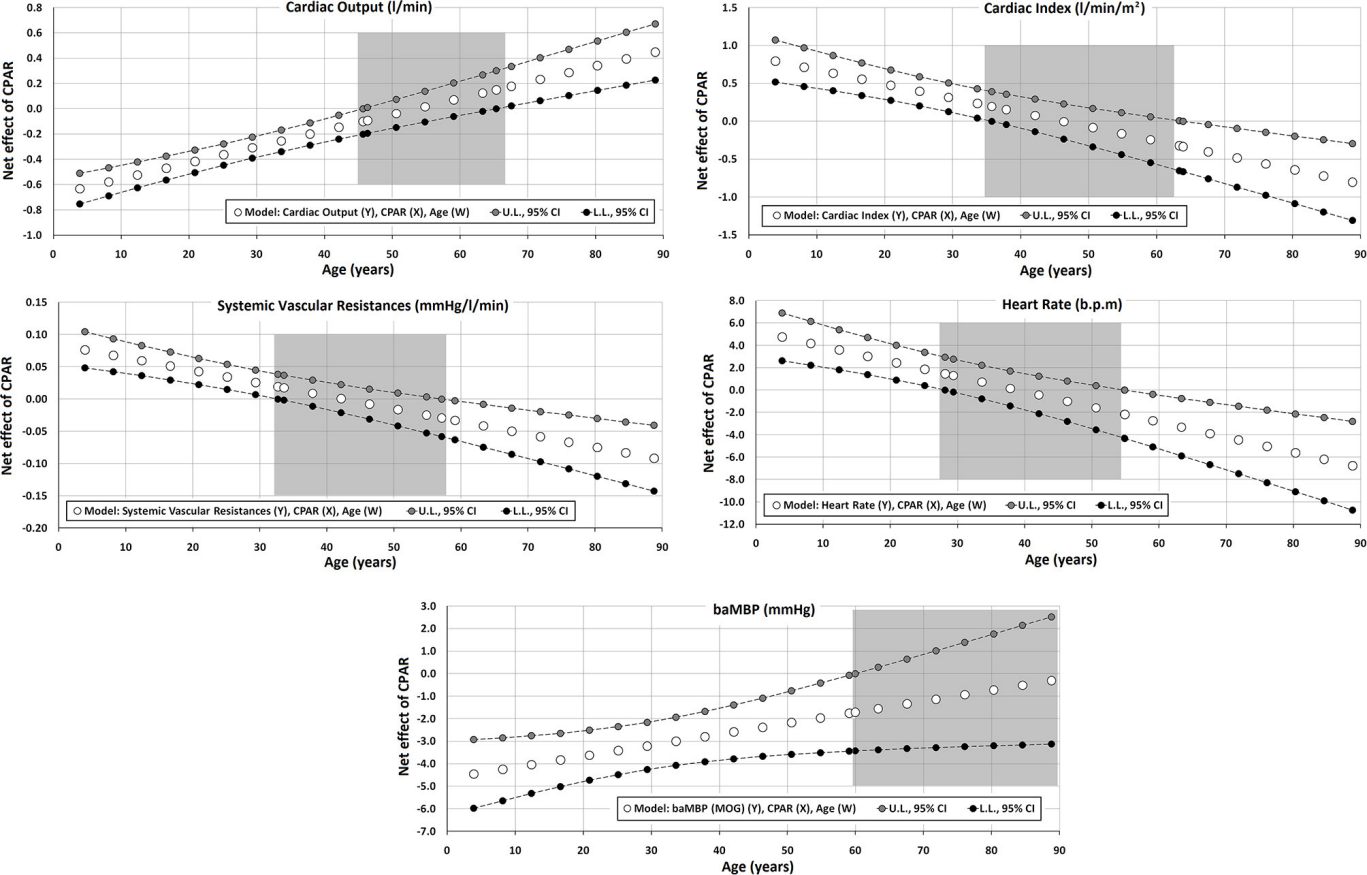
Effect of compliance with physical activity recommendation (CPAR) condition on global hemodynamic indexes: effects at different ages throughout life. The gray area shows regions that do not reach statistical significance. Complete quantitative information related to interactions and/or Johnson-Neyman regions is detailed in Supplementary Tables 2–95. In each panel, the “net or absolute conditional effect” (and its 95% confidence interval) of reaching CPARs = 1, quantified in the units of the cardiovascular variable, was plotted as a function of age. If the confidence interval crosses the value “0,” the effect of CPARs on the cardiovascular variable becomes statistically non-significant (*p* ≥ 0.05).

**Figure 3:**
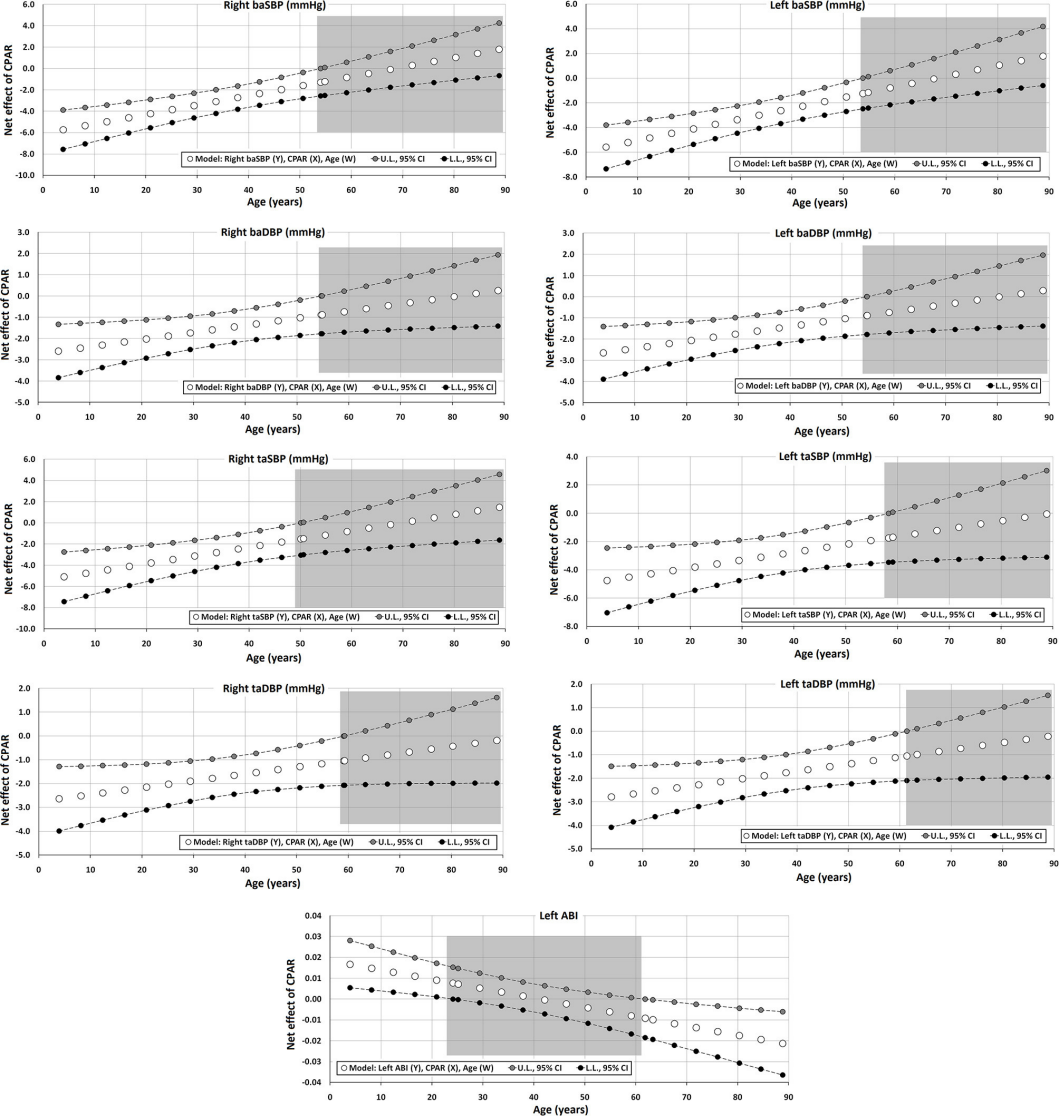
Effect of CPAR condition on brachial and tibial artery blood pressure and brachial-ankle index: effects at different ages throughout life. The gray area shows regions that do not reach statistical significance. Complete quantitative information related to interactions and/or Johnson-Neyman regions is detailed in Supplementary Tables 2–95. In each panel, the “net or absolute conditional effect” (and its 95% confidence interval) of reaching CPARs = 1, quantified in units of the cardiovascular variable, was plotted as a function of age. If the confidence interval crosses the value “0,” the effect of CPARs on the cardiovascular variable becomes statistically non-significant (*p* ≥ 0.05).

**Figure 4:**
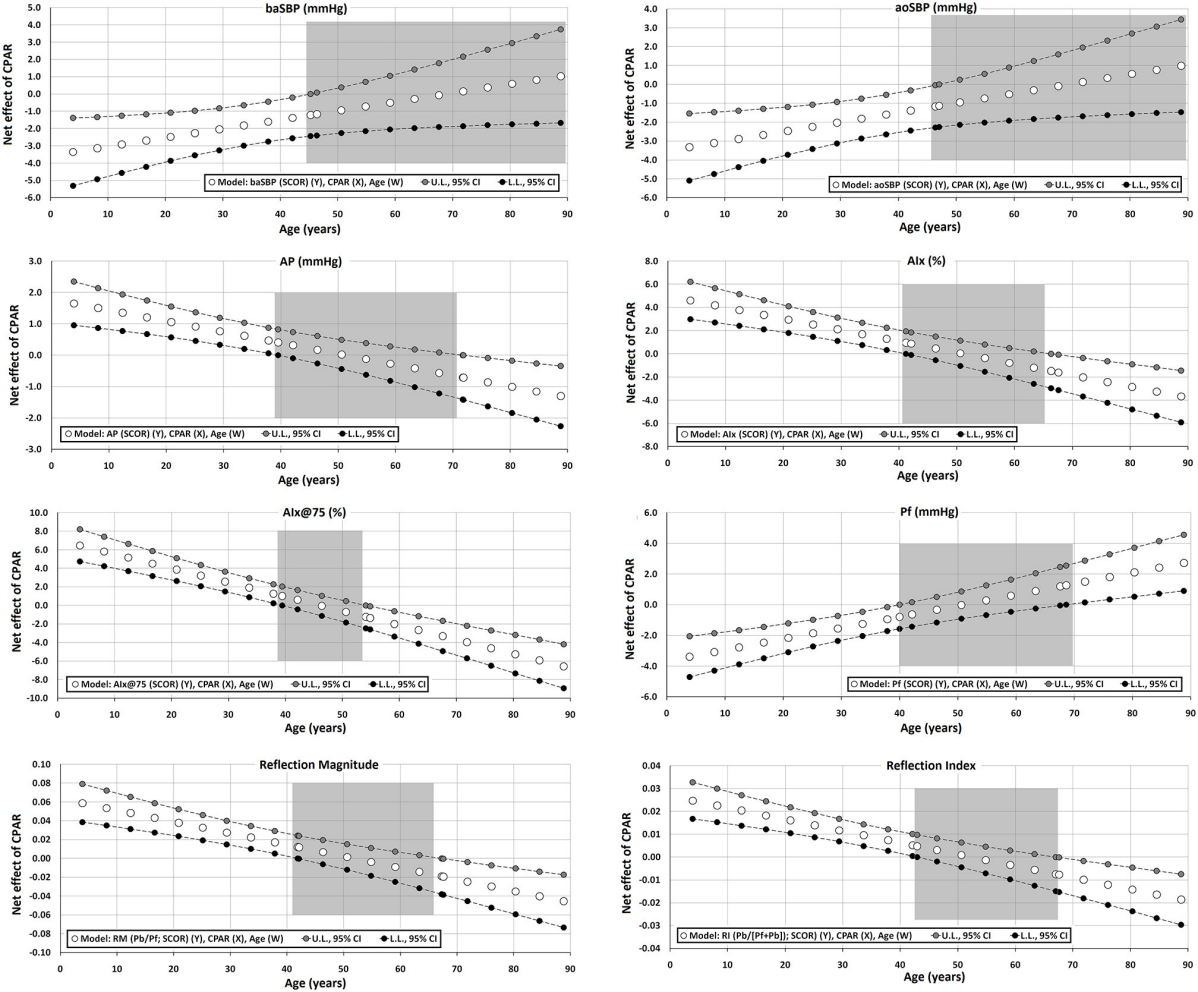
Effect of CPAR condition on aortic blood pressure levels and waveform-derived indexes: effects at different ages throughout life. The gray area shows regions that do not reach statistical significance. Complete quantitative information related to interactions and/or Johnson-Neyman regions is detailed in Supplementary Tables 2–95. In each panel, the “net or absolute conditional effect” (and its 95% confidence interval) of reaching CPARs = 1, quantified in units of the cardiovascular variable, was plotted as a function of age. If the confidence interval crosses the value “0,” the effect of CPARs on the cardiovascular variable becomes statistically non-significant (*p* ≥ 0.05).

**Figure 5:**
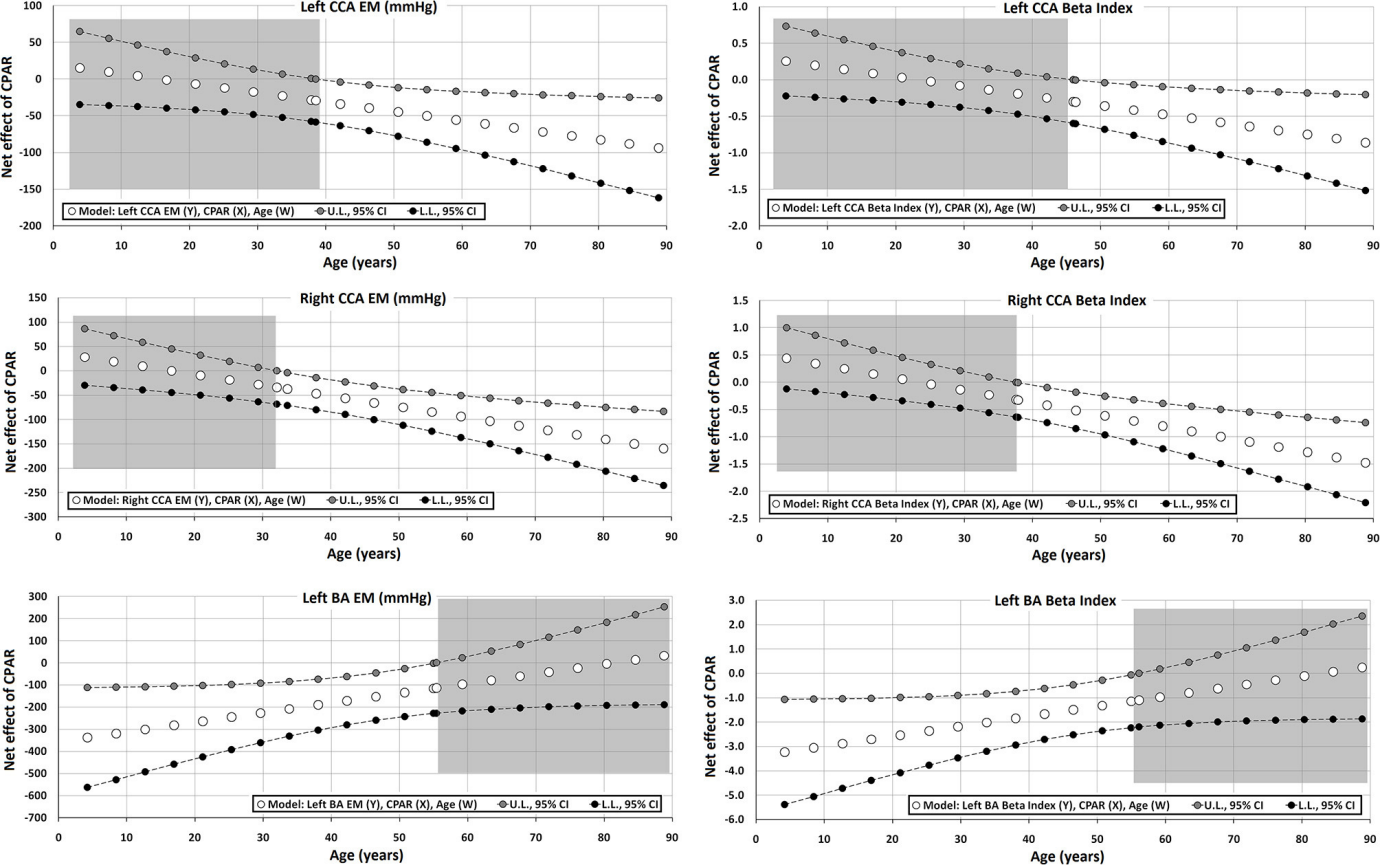
Effect of CPAR condition on local arterial stiffness: effects at different ages throughout life. The gray area shows regions that do not reach statistical significance. Complete quantitative information related to interactions and/or Johnson-Neyman regions is detailed in Supplementary Tables 2–95. In each panel, the “net or absolute conditional effect” (and its 95% confidence interval) of reaching CPARs = 1, quantified in the units of the cardiovascular variable, was plotted as a function of age. If the confidence interval crosses the value “0,” the effect of CPARs on the cardiovascular variable becomes statistically non-significant (*p* ≥ 0.05).

**Figure 6:**
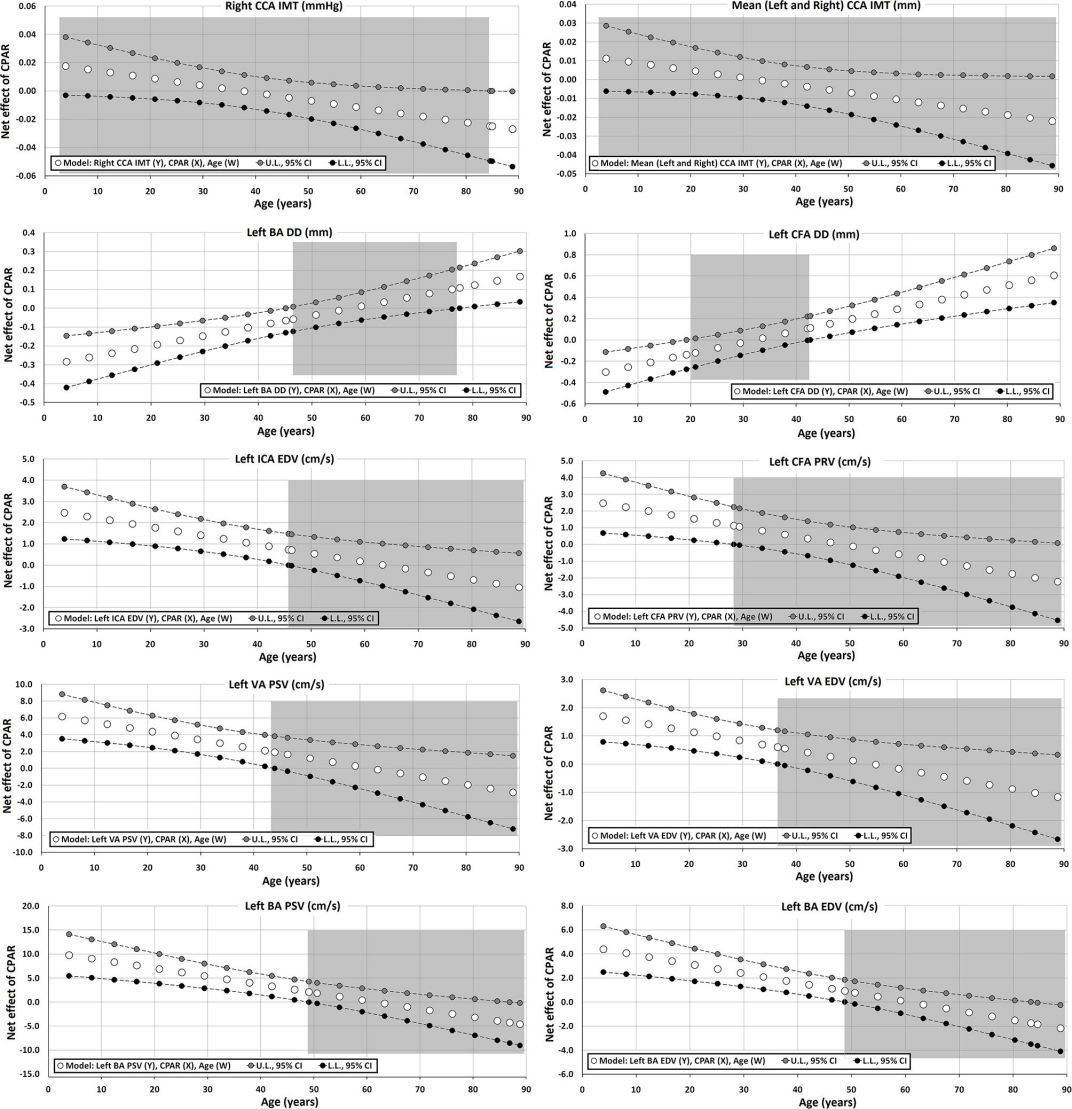
Effect of CPAR condition on arterial structural (intima-media thickness and arterial diameters) and blood flow velocity levels: effects at different ages throughout life. The gray area shows regions that do not reach statistical significance. Complete quantitative information related to interactions and/or Johnson-Neyman regions is detailed in Supplementary Tables 2–95. In each panel, the “net or absolute conditional effect” (and its 95% confidence interval) of reaching CPARs = 1, quantified in units of the cardiovascular variable, was plotted as a function of age. If the confidence interval crosses the value “0,” the effect of CPARs on the cardiovascular variable becomes statistically non-significant (*p* ≥ 0.05).

The Johnson-Neyman technique was used to produce regions of significance in the multivariate models, but only when the corresponding interaction (CPAR^*^age) has a *p* ≤ 0.1. This analysis allowed to evaluate the conditional effect of CPARs (x) on cardiovascular properties (y) at different ages (moderator, w) (Hayes, 2020). The moderation effect was graphically depicted in Figures 2–6. Complete quantitative information related with interactions and/or Johnson-Neyman regions is detailed in Supplementary Tables 2–95. In each figure, the “net or absolute conditional effect” (and its 95% confidence interval) of reaching the PA recommendations (CPARs = 1), quantified in units of the cardiovascular variable, was plotted as a function of age. In each figure, if the confidence interval crosses the value “0,” the effect of CPARs on the cardiovascular variable becomes statistically non-significant (*p* ≥ 0.05).

#### Sample Size and Statistical Package

According to the central limit theorem, Kurtosis and Skewness coefficient distribution, and number of studied subjects (with sample size >30), a normal distribution was considered (Lumley et al., 2002). Different methods have been described to calculate sample size for multiple linear regression. The number of subjects included (*n* = 3,619) was higher than the minimum sample size required to perform an extremely rigorous and conservative multivariate linear regression analysis, considering significance level (α): 0.01, power: 0.9, predictors (independent variables): 16, and a very reduced effect size: 0.1 (included: 3,619, minimum required sample size: 3,152). Similarly, the sample size is also noticeably greater than the minimum necessary when it is calculated with other traditional methods (e.g., Green's rule of thumb [medium effect] to test an entire model [*n* = 50 + 8^*^predictors = 178] or coefficients [*n* = 104 + predictors = 120]). Consequently, our approach allows us to achieve very robust results with low uncertainty from the point of view of confidence intervals reached.

When analyzing and discussing the results, the following operational definitions were used: (i) childhood (up to 10 y), (ii) adolescence (10–22 y), (iii) and adulthood (22 y and older). Adult life was divided into four stages: early adulthood (22–34 y), early middle age (35–44 y), late middle age (45–64 y), and late adulthood (65 and older) (Medley, 1980; Hochberg and Konner, 2020).

Analyses were performed using SPSS Software (v.26; IBM-SPSS Inc., Chicago, IL, United States). PROCESS version 3.5 (SPSS extension) was used for moderation (interaction) and Johnson-Neyman analysis (Hayes, 2020). *p* < 0.05 was considered statistically significant.

## Results

### Characteristics of the Subjects

Table 1 shows the characteristics of the subjects. Note the balanced sex distribution and wide ranges of age and CRF levels (e.g., HDL cholesterol: 14–109 mg/dl). Note that subjects who met the WHO-related PA recommendations (CPAR Group) had lower CRF exposure levels compared to subjects included in the non-CPAR group.

Supplementary Table 1 presents a comprehensive characterization of the cardiovascular system including 96 cardiovascular variables. Notably, there were subjects with very different levels of cardiovascular characteristics, which strengthened our association analyses (e.g., baSBP between 89 and 195 mmHg, CO between 3.5 and 7.1 L/min, cfPWV between 2.95 and 19.75 m/s, ABI between 0.68 and 1.61, and CCA DD between 3.73 and 11.04 mm).

### Compliance With Physical Activity Recommendations and Cardiovascular Risk Factors

Regardless of age, sex, and use of antihyperlipidemic and antidiabetic drugs, CPARs was significantly associated with (i) lower levels of BW, BMI, total and LDL cholesterol, atherogenic index, triglycerides, glycemia, and creatinine, and (ii) higher levels of HDL cholesterol.

In all the cases, the results obtained directly in our sample were endorsed by Bootstrapping analysis (Table 2).

### Compliance With Physical Activity Recommendations and the Cardiovascular System

#### Global Hemodynamic State

CPAR status was independently associated with variations in global hemodynamic parameters, in which age moderated this relationship (Table 3, Figure 2). In the first 3–4 decades of life, younger ages were associated with (i) lower CO and baMBP and (ii) higher SVR, HR, and CI. Conversely, In subjects over 55–65 y, with increasing age, CPAR status was associated with (i) lower CI, SVR, and HR, and (ii) higher CO. In those over 60 y, CPAR status was not associated with variations in baMBP levels.

#### Aortic, Brachial, and Tibial Pressures and Ankle Brachial Index

Regardless of other CRFs, CPAR status was significantly associated with lower DBP and SBP of both the brachial and tibial arteries. However, while this effect was markedly evident in subjects with decreasing age, it was no longer significant between 50 and 60 years of age (Table 4, Figure 3). Of note, this age-moderated association results in an average decrease in right baSBP of 6, 5, 4, 3, and 2 mmHg in subjects 4, 12, 20, 33, and 50 years of age, respectively (Supplementary Table 7). The results observed on the left and right hemibodies were similar for the BP measurements in the brachial and tibial arteries.

Moreover, in both Table 5 and Figure 4, it can be observed that aoBP levels are also lower in subjects with CPAR status and <45 years of age. In this case, this age-moderated association results in an average decrease in aoSBP of 3, 3, 2, and 2 mmHg in subjects 4, 12, 20, and 33 years of age, respectively (Supplementary Table 20).

Finally, CPAR status was associated with the left ABI (left taSBP/baSBP ratio), in which younger subjects demonstrated higher CPAR-related levels, and subjects older than 60 y showed lower levels (Figure 3).

#### Central Aortic Wave-Derived Parameters

Regardless of the other CRFs, CPAR status showed an age-moderated association with different aortic pressure waveform-derived indexes. Particularly in younger subjects, CPAR status was associated with (i) lower Pf levels (without being associated with Pb levels) and consequently with (ii) higher AP, AIx, AIx@75, RM, and RIx levels (Table 5, Figure 4).

Of note, in those who were between 40 and 65 years of age, the association with CPAR status was non-significant. In these subjects, however, Pf levels associated with CPAR status increase gradually with increasing age, while the opposite was observed for AP, AIx, AIx@75, RM, and RIx levels (Figure 4). This condition would be related to higher CO and reduced SVR state, similar to the one observed in subjects with CPAR status with increasing age (Figure 2). In this setting, higher Pf (associated with higher CO) and lower wave reflection levels (associated with lower SVR) would result in lower AP, AIx, AIx@75, RM, and RIx among subjects with CPAR status (Table 5, Figure 4).

#### Local Arterial and Regional Arterial Stiffness, and Stiffness Gradient

CPAR status did not show a significant association with regional arterial stiffness levels (cfPWV and crPWV) or stiffness gradient (PWV ratio) (Table 6).

However, in adults, CPAR status was independently associated with lower left and right CCA stiffness (CCA EM and β), and the associated reduction was higher with increasing age (Table 7, Figure 5). This association started to be notable from 30 to 40 and 40 to 50 years of age for the right and left CCAs, respectively. In addition, in subjects older than 50–60 y, the gradual increase in brachial stiffness (BA EM and β) associated with CPARs that began in childhood was no longer observed.

#### Macro- and Micro-Vascular Reactivity Indexes

CPAR status was not independently associated with any of the vascular reactivity indexes (Table 8).

#### Arterial Diameter, Intima-Media Thickness, Blood Flow Velocity, and Doppler Indexes

Regardless of the other CRFs, CPAR status was not clearly associated with IMT levels, although older age was associated with lower right CCA IMT levels (Table 9, Figure 6).

Carotid diameters were not associated with CPAR status (Table 9). Conversely, although only for the left hemibody, CPAR status was associated with higher CFA DD and BA DD levels in adult subjects.

In younger subjects, CPAR status was associated with higher blood flow velocities (e.g., left ICA EDV, VA PSV, and BA PSV), a finding that was progressively minimized until approximately the age of 40, where it was no longer significant (Table 10, Figure 6). CPAR status was not associated with Doppler indexes such as RI and PI (Table 11).

## Discussion

This study sought to evaluate to what extent the independent association between CPARs of the WHO and the hemodynamic, structural, and functional characteristics of the arterial system was moderated by the age of the subjects. To this end, we designed and carried out a large sample-size population study that included a wide age range composed of children, adolescents, adults, and the elderly. After analyzing 3,619 subjects, the main results of this study are summarized and analyzed in the following sections.

### Age as a Moderating Variable of the Association Between CPARs and the Arterial System

First, the association between CPAR status and different cardiovascular characteristics was independent of other traditional CRFs, and, at the same time, was significantly moderated by age. The “age-dependent moderation” of CPAR status was observed for a wide age range (early childhood to late adulthood), but particularly notorious for the extremes of life, and for different hemodynamic, structural, and functional properties of the arterial system. Moreover, certain arterial characteristics demonstrated opposite effects in relation to CPAR status depending on the age range considered.

For instance, there were vascular variables, such as BA PSV and ICA EDV, that were associated with CPAR status only in the first 3–4 decades of life (i.e., childhood, adolescence, early adulthood, and early middle age ranges), or that were associated with CPAR status only partially excluding “late adulthood” (e.g., aoBP and baBP). While other associations, such as with CCA EM, were only notable after the “early adulthood” stage, and other variables (i.e., SVR, RM, Pf) were only significantly associated with CPAR status in the early and late stages of life (e.g., from childhood to early adulthood, and finally “late adulthood”), but not during intermediate adult ages.

Strikingly, certain vascular variables, such as regional arterial stiffness and vascular reactivity, did not show any significant association with CPAR status. Consequently, PA-related effects on the cardiovascular system, with its local and regional disparities, may express or stop expressing gradually throughout the growth and aging processes. Nevertheless, at least in theory, the “loss and emergence” of any association between CPAR status and vascular characteristics throughout life could be influenced by prior associations, regardless of if they were or were not appreciated before. For instance, the low aoBP levels observed in younger subjects of the CPAR group, which were no longer appreciated in subjects older than 45 y (Figure 3), could be responsible for the CPAR-related low CCA stiffness observed in advanced age (Figure 5).

The moderating effect of age on the relationship between PA and vascular characteristics was analyzed, although partially, in prior studies (Vaitkevicius et al., 1993; Tanaka et al., 2000, 2002; Moreau et al., 2006; McDonnell et al., 2013; Shibata et al., 2018). For instance, one of these reports pointed out that the “greatest impact” or beneficial effects of PA on the vascular system were observed in the smaller pre-resistance and resistance vessels in younger subjects and in the large elastic arteries in older subjects (McDonnell et al., 2013). Our results are only partially in agreement with this postulation. On one hand, we found that (i) “large elastic arteries” (e.g., CCA stiffness), were only associated with CPAR status in subjects older than 40 y, and that (ii) BP levels and diastolic blood flow velocities of the ICA, VA, and BA (related with global and local peripheral resistances) were only associated with CPAR in young adults. On the other hand, the analysis of other related variables describing “smaller pre-resistance and resistance vessel characteristics” (e.g., SVR, Doppler indexes) does not allow us to confirm entirely the prior postulation. Moreover, the association between CPAR status and large transitional (BA) and muscular (CFA) arterial diameters and stiffness precluded a clear differentiation between younger and older subjects. Given that our approach included data of a larger set of parameters characterizing more arterial sites and regions, we observed that the relationship between CPAR and large arteries is likely more complex and arterial region/site-dependent.

Interestingly, CPAR status was associated with “opposite effects” on some arterial parameters in younger and older subjects. For instance, in contraposition to what was observed in older subjects, CPAR status in young individuals was associated with higher AP, RM, and RIx (Figure 4). Conceptually speaking, this observation agrees with that of other authors. For instance, McDonnell et al. (2013) reported that while baPP and aoPP were elevated in younger subjects who undertook regular PA, these variables were lower in older active subjects compared to age-matched sedentary subjects. Thus, it is clear that the relationship between CPAR status and the arterial system is complex therefore, any generalization of the association between PA and arterial properties might easily lead to inaccuracies if the age or stage of life of the subject is not considered.

### From “Early to Late” Adulthood: Age-Related Moderation of the Association Between CPARs and the Arterial System

Second, with increasing age, in adults, the CPAR status demonstrated an increasingly beneficial hemodynamic profile (in relative terms). Although no significant changes were observed in aoBP or baBP, the main static (e.g., SVR) and dynamic (e.g., central arterial stiffness) left ventricle afterload determinants were significantly lower. Moreover, throughout adulthood, CPAR status was associated with lower SVR, HR, carotid stiffness, absolute, and relative contribution of wave reflection to pulse pressure waveform (AP, AIx@75, RM, and RIx), longer arterial diameters (lower impedance) of the peripheral arteries (CFA and BA DDs) and higher CO (Figures 2, 4–6). The fact that the increased CO occurred in the setting of lower HR levels points to an improvement in ventricular systolic function.

Therefore, in this study, the relationship between CPAR and vascular properties observed in adults might be also consistent with a CPAR-related longer-term reduction in cardiovascular risk. In this regard, Vaitkevicius et al. (1993) reported that endurance-trained men (*n* = 14, ≥54 y) had significantly lower AIx and cfPWV levels (36 and 26% lower, respectively) than their sedentary age peers (*n* = 146, age mean/range: 54/21–96 y) despite similar baSBP and baPP levels. Although this study evaluated senior athletes and not the general population, our results agree that in subjects older than 55 years, higher PA was associated with lower AIx and arterial stiffness of the large central arteries. Of note, while the authors found lower regional stiffness levels (i.e., cfPWV), they found lower local (i.e., CCA EM and β) but not regional arterial stiffness. It is possible that the PA performed by our population of subjects did not reach the threshold to induce a drop in general arterial stiffness able to be detected with regional arterial markers. Likewise, the authors did not find any significant association between PA and lower baBP (Vaitkevicius et al., 1993). Tanaka et al. (2000) also reported a meaningful role of age in moderating the relationship between PA and CCA stiffness, and this effect was “dose-dependent.” These authors measured CCA stiffness (in terms of compliance and β) in 151 healthy normotensive and non-obese men (age: 18–77 y) assigned to one of three groups: (i) “sedentary” (*n* = 54; no-regular PA), (ii) “recreationally active” (*n* = 45; light-to-moderate PA ≥ 3 times/week), and (iii) “endurance exercise–trained” (*n* = 53; vigorous aerobic endurance PA ≥ 5 times/week and active in local road running races), and grouped in age-related subgroups: young (18–37 y), middle-aged (38–57 y), and older (58–77 y). Although not statistically significant, the authors observed that CCA compliance in middle-aged and older recreationally active men were 10–17% greater, respectively, than in their sedentary peers, again pointing to an age-related moderation effect of PA. Moreover, even adherence to “light PA” may have a small but potentially physiologically relevant effect on CCA stiffness among adult individuals (Tanaka et al., 2000). This study also revealed that with increasing age, those subjects that remained physically actives demonstrated lower elevation of CCA stiffness associated with aging. In fact, differences in CCA compliance between the young and older groups of endurance-trained men were reduced (25%) compared to the differences observed between the young and older groups of sedentary men of (45%) (Tanaka et al., 2000). As observed in our study (Figure 5), changes in CCA stiffness were not associated with PA in the young adults (18–37 y) (Tanaka et al., 2000). In a cross-sectional study on 83 healthy non-smoker subjects (age: 18–78 y) separated into two groups of “less active” (light-to-moderate PA ≤ 3 times/wk) and “highly active” (vigorous PA ≥5 times/wk) subjects, Holland et al. observed a blunted age-associated increase in arterial stiffness (in subjects aged ≥40 y), in particular among highly active subjects. In other words, the expected aging-related increase in arterial stiffness can be particularly attenuated by vigorous activity (Holland et al., 2017). Of note, in this study, both groups were reported, although subjectively, to have maintained the specified PA level for at least the past 5 years.

On the other hand, McDonnell et al. examined the age-dependent relationship between regular PA and aortic stiffness and wave reflections in a large group of healthy non-smoker individuals (*n* = 1,036): (i) younger (<30 y, mean/SD: 21 ± 5 y) and (ii) older (>50 y, mean/SD: 6.3± 7 y) who were either sedentary or undertook a regular aerobic PA (McDonnell et al., 2013). In the older than 50-y-old subjects, regular PA was associated with lower baBP, aoSBP, HR, and aortic stiffness (cfPWV). The BP reduction found by McDonnell et al. would be consistent with our study, since in our population, until the age of 60 years, there would be a PA-related reduction in BP (Figure 3). Moreover, the older group of active subjects of the aforementioned study was associated with lower HR and a trend of presenting lower SVR and higher CO, which is again similar to our findings (McDonnell et al., 2013). Finally, in consonance with our results, the authors reported a non-significant association between levels of PA and AIx or AP. Unfortunately, the data presented by the investigators precluded further analyses of older age, in particular whether the elderly subjects would begin to express differences in AIx and AP associated with PA, which is in line with our study (Figure 4).

More recently, Shibata et al. (2018) performed a cross-sectional examination of 102 seniors (> 60 y, age mean/SD: 70/6 y) who had a consistent lifelong exercise history. Based on exercise frequency (as an index of exercise “dose”), the subjects were stratified into four groups: (i) sedentary (<2 sessions/week), (ii) casual exercisers (2–3 sessions/week), (iii) committed exercisers (4–5 sessions/week), and (iv) master athletes (6–7 sessions/week and regular competitions) (Shibata et al., 2018). Like in our group of subjects >60 y, the investigators reported that PA was not associated with lower baBP but with less HR and a trend of having lower AP, Aix, and AIx@75 when comparing sedentary vs. casual exercisers and/or committed exerciser subgroups. Similarly, the authors also reported that CCA β (but not cfPWV) was higher in sedentary seniors compared to the other groups. In addition, crPWV and femoral-dorsal (lower limb) PWV, that it is to say, “upper and lower limb peripheral PWVs,” were not significantly different among the groups (Shibata et al., 2018). This supports the notion that the impact of PA on arterial stiffness is particularly notable in the central arteries (e.g., CCA).

Regardless of age, we found no association between CPAR status and CCA IMT or CCA diameter (Figure 6). In this regard, Tanaka et al. evaluated the CCA IMT and IMT-to-lumen ratio in 137 healthy, normotensive, and non-obese men (age: 18–77 y) who were either (i) sedentary (no regular PA) or (ii) endurance-trained (vigorous endurance exercise >5 times/week and active in local road running races) (Tanaka et al., 2002). The study showed that the CCA IMT and IMT/lumen ratio did not change with the PA “intervention” (Tanaka et al., 2002). Likewise, in 432 healthy subjects (age mean/range: 43/30–60 y), Kozakova et al. (2007) found that CCA stiffness, but not the IMT, was negatively correlated to magnitude and PA patterns (assessed by accelerometry) independently of other CRFs.

Although these findings support no association of arterial wall thickness with PA, it might depend on the artery evaluated, or alternatively, on the magnitude or frequency of PA. In an interesting study, Moreau et al. measured the right CFA IMT in 173 healthy men and 74 healthy women (non-smokers, free of main CRFs) in relation to three groups of PA level: (i) sedentary (no regular PA), (ii) moderately aerobically active (light-to-moderate intensity PA ≥3 times/week), and (iii) endurance exercise-trained (vigorous aerobic endurance PA ≥5 times/week and competing in local road running races), young (20–39 y), middle-aged (40–59 y), and older (60–79 y) men. Accordingly, while the investigators found that the CFA IMT increases with age even in habitually exercising adults, the age-associated increase and absolute level of CFA IMT were smaller in middle-aged and older adults who perform regular aerobic-endurance exercise. The authors hypothesized that these findings may explain the lower incidence of atherosclerotic disease observed in physically active subjects. Although we found no significant association between CPAR status and CFA IMT (or with any of the other arteries evaluated), we did observe a structural association with CFA. The CFAs “became larger” in association with CPAR, an observation that was enhanced with increasing age (Figure 6). All the data analyzed together reflect a potential beneficial effect of PA, likely in relation to better local blood flow conditions, local endothelial function, and reduced atherosclerosis risk.

In other words, considering the differential associations between PA (in our study population mainly aerobic exercise) and CCA and CFA territories, it is possible that these disparities could be explained by PA itself (“stimulus”). On one hand, the lack of PA-related effects on CCA IMT and diameter may be due to the apparent inability of habitual exercise to prevent or reduce the age-associated elevation in aoBP (Tanaka et al., 2002). On the other, most aerobic-endurance exercise is performed primarily with the legs, resulting in regular (e.g., daily) and sustained elevations in CFA blood flow. In turn, elevations in blood flow would induce an increase in CFA shear-stress and nitric oxide bioactivity, thus (i) increasing CFA DD, resulting in less “local impedance” and consequently (ii) reducing CFA peak reversal velocity (Figure 6) and possibly (iii) suppressing or attenuating the mechanisms contributing to age-associated increases in CFA IMT, as found by others (Moreau et al., 2006).

### From “Childhood” to “Early Adulthood:” Age-Related Moderation of the Association Between CPARs and the Arterial System

Third, in subjects younger than 45–55 y of age (childhood, adolescence, and early middle age) and particularly with decreasing age, CPAR status was associated with lower aoBP, baBP, and taBP (Figures 3, 4). Lower BP levels could be explained by lower Pf, which in turn could be associated with the observed lower CO values associated with younger ages (Figures 2, 4). In parallel, indexes that assess wave reflection contribution to the BP waveform (e.g., AIx@75, RM y, and RIx) were found to increase in relation to CPAR status and decreasing age (Figure 4).

An initial evaluation of these results could sound theoretically contradictory. Although a lower aoSBP associated with CPAR status could be interpreted as beneficial (i.e., low left ventricle afterload), a higher magnitude of wave reflection is usually associated with higher LV afterload. Although future studies are needed to better clarify this concept, it seems that the higher relative contribution of wave reflections to pressure waveform were not determined by increase in Pb rather than decrease in Pf, which in turn would lead to higher levels in AIx, RM, and RIx. Importantly, these results derived from pulse wave and wave separation analyses occurred in the setting of a reduction in aoSBP and aoDBP associated with CPAR status and decreasing age. Of note, aoBP is the real determinant of left ventricular afterload, as this is the pressure that the left ventricle must work against to eject blood during systole. Consequently, the increase in wave reflection indexes (i.e., AIx and AIx@75) observed in association with CPARs in young individuals could lead to inaccurate s of increased afterload, when in fact the opposite might be true.

As mentioned before and consistent with our results (Figure 5), Tanaka et al. (2000) reported no association between variations in CCA stiffness and PA levels in young adults (18–37 y). On the other hand, McDonnell et al. (2013) found that regular exercise in younger subjects (<30 y, 21 ± 5 y) was associated with lower baDBP and aoSBP but unchanged aortic stiffness (cfPWV).

However, unlike our study, the investigators observed that regular exercise was associated with lower HR, SVR, Aix, and AP but unchanged baSBP, CO, and CI (McDonnell et al., 2013). Given that these results are consistent with our findings but in older individuals, it is possible that their results may represent anticipated effects of PA on the arterial system in relation to higher levels of PA than just CPARs.

### CPARs and Cardiovascular Risk Factors

Finally, we found that regardless of age, sex, and use of antihyperlipidemic and antidiabetic drugs, CPAR status was significantly associated with (i) lower levels of BW, BMI, and glycemia, (ii) better lipid profile (higher LDL, lower total and LDL cholesterol levels, atherogenic index, and triglycerides), and (iii) lower creatinine serum levels.

The link between PA and better CRF profile has been consistently observed in different studies performed on adults. In fact, PA remains the cornerstone of non-pharmacologic treatment for patients with metabolic syndromes in international guidelines (World Health Organization, 2019, 2020). Undoubtedly, a rigorous study on the association between PA and vascular properties requires adjusting for other CRFs to assess its independent association, not only because the presence of CRFs might induce confounding bias, but also because the beneficial effect of PA on arterial properties may be facilitated/enhanced by parallel improvement in CRFs achieved by the exercise itself.

Similar results have been previously reported by other investigators (Moreau et al., 2006; McDonnell et al., 2013). Moreau et al. (2006) found a significant association between PA levels and lower BW, LDL-cholesterol levels, and fasting insulin levels, with the greatest effect being observed in endurance-trained men. However, McDonnell et al. showed no significant differences in BW, BMI, or waist circumference when comparing “active” vs. “sedentary young individuals” (<30, 21 ± 5 y). A meaningful association between PA and anthropometric measures was only seen in older subjects (> 50 y; age: 63 ± 7 y) (McDonnell et al., 2013). Nevertheless, in general, these investigators found that irrespective of age, higher PA was associated with better biochemical profiles such as lower triglycerides and glucose and higher HDL cholesterol levels.

### Physiological and Practical Implications

The following physiological implications and/or practical applications could be attributed to this study and its findings: (i) contribution to the generation of hypothesis regarding considerations to be taken into account when selecting parameters to assess CPAR impact on the vascular system, and, as discussed, (ii) contribution to the knowledge and understanding of links (associations) between CPARs and status of the vascular system, and how they could be moderated by age.

Regarding the former, looking at our findings, it could be proposed that the independent relationship between CPARs and cardiovascular status could be mainly evaluated (“visualized”) by determining: (i) baBP or aoBP levels (in subjects <50–55 y) and (ii) aortic waveform-derived indexes (in subjects >50–55 y). At present, the abovementioned recordings and parameters can be obtained simultaneously by laboratory and ambulatory operator-independent evaluations using a single device (e.g., Sphygmocor, Mobil-O-Graph). Of note is that BP and waveform-derived parameters would allow for assessment of cardiovascular status in association with CPARs, in a broad range of age (3–90 years), with independence of subjects' exposure to other CRFs. Then, by quantifying those parameters, researchers and/or clinicians could evaluate the independent impact of CPARs on the vascular system whatever age group is considered.

Our results also showed that some parameters deemed useful to assess cardiovascular status in research or clinical practice, enabling to evaluate hemodynamic (e.g., Doppler indexes), functional (e.g., vascular reactivity, regional stiffness), or structural (e.g., CCA IMT) properties, did not show an independent association with CPARs, or that it was not clear (e.g., it was only observed for a hemibody). Then, although those parameters could be useful when analyzing the impact of a given PA intervention on specific cardiovascular properties, they would not be considered as elective indexes (i.e., first choice) if the aim is to select sensitive indicators of easy measurement to study the relationship between CPARs and the cardiovascular system. In other words, looking at our findings, it could be said that in this context, it would not be strictly necessary to perform a multiparametric approach (e.g., determination of hemodynamic, structural, and functional arterial characteristics), since data provided by BP and waveform-derived indexes would be enough for the objectives pursued.

Regarding the second point, our results showed that in the first decades of life, especially during childhood and adolescence, CPARs would be associated mainly with hemodynamic variations, which would result in reduced maximum (systolic) and minimum (diastolic) BP levels. At older age, associations between CPAR and BP (systolic and diastolic) were no longer observed, but arterial changes associated with CPARs began to be noted in waveform-derived indexes. Thus, at older age, changes in waveform-derived indexes would allow for assessment of the independent impact of CPARs on cardiovascular properties that would be the result of biological changes occurring at different levels. In this regard, CPARs would be associated with changes in structural and functional arterial properties (e.g., larger diameters and lower stiffness levels), as well as in left ventricular function. Although these variables, in isolation, would not be good markers to assess the impact of CPARs on the cardiovascular system, their changes could contribute to variations in waveform-derived indexes (e.g., AP, AIx@75). In turn, these would be considered appropriate markers of the impact of CPARs on the cardiovascular system (“centering or summarizing” the effects on other cardiovascular properties).

Finally, it is of note that variables less sensitive to CPARs showed differences in their behavior in terms of association with CPARs. About this, in agreement with previous studies (already discussed) we found that central elastic arteries would be the vessels most sensitive to lifestyle factors related to PA.

### Strengths and Limitations

This study has strengths and limitations that should be considered. First, our study included a comprehensive non-invasive evaluation of cardiovascular properties obtained from a large population sample of children, adolescents, and adults (3–90 y). Consequently, the range of age was very wide. Thus, a robust analysis in terms of age-related moderation was possible. The wide range of age and large sample size of the study (*n* = 3,619) allow us to assess age as a continuous moderating variable, without the need for simplifying or reducing the analysis to simple group comparisons [e.g., comparing young [<30 y] vs. older [>50 y] individuals] (Tanaka et al., 2000; McDonnell et al., 2013; Holland et al., 2017; Majerczak et al., 2019). The inclusion of children, adolescents, and adults allow us to assess almost the entire lifespan of the individuals; therefore, we can assess whether changes found in one stage of life are specific or progressive. Second, we evaluated a large set of arterial parameters (i.e., 96 variables of hemodynamic, structural, and functional properties) assessing different histological types of arteries from different sites (central vs. peripheral). By obtaining these data, we were able to compare the association between CPAR status and the arterial system, analyzing potential differences in impact that PA could have depending on arterial territory considered. To the best of our knowledge, there are no studies of this type in the literature. Additionally, in this study, aortic and brachial BPs were used to quantify central and peripheral arterial stiffness levels, respectively. This should be considered a strength, since previous studies have quantified CCA stiffness using baPP (Kozakova et al., 2007), which could lead to inaccuracies of parameters. We are not proposing that all these parameters should be measured when evaluating the impact of PA on the cardiovascular system. In fact, our results evidence that it would not be strictly needed to perform an approach that implies simultaneous determination of hemodynamic, structural, and functional arterial characteristics, as the information provided by BP levels and waveform-derived indexes would be enough to evaluate the impact of CPARs on the vascular system in the general population (3–90 y).

Third, regarding cardiovascular analyses, whenever possible, we decided to analyze each hemibody individually. Although this increases the complexity of the assessment, as it increases the number of measurements, and significant differences can only be found in one hemibody. However, this better represents the reality, since either averaging both sides or studying only one hemibody will prevent us from being able to assess already known anatomic and functional variations between hemibodies. Fourth, sex was included in the regression models as a cofactor. Accordingly, we ensured that sex was not globally influencing the results. Furthermore, we analyzed the association between CPAR status and arterial properties regardless of anthropometric characteristics, CVD, CRFs, and pharmacologic treatment. This allows us to study the independent association between CPAR status and the arterial system. Unfortunately, this was not performed by others (McDonnell et al., 2013; Majerczak et al., 2019).

Finally, no subjects were excluded in this study based on PA. In this way, each subject was classified in one of the two possible groups: CPARs = 1 (yes) or CPARs = 0 (no). This differs from other studies that excluded subjects “to ensure a clear separation of the groups in terms of the amount of PA” [e.g., subjects who undertook regular resistance training and/or low-intensity PA or individuals who took part in a mixture of resistance and aerobics PA were excluded (McDonnell et al., 2013)]. When excluding subjects with intermediate levels of PA and controlling for the study, assessment of the general population (“real world”) is no longer possible and might create an artificial amplification of differences in time of comparing two “extreme groups” of subjects clearly sedentary vs. those with high levels of PA. To avoid this possibility, our approach aimed at classifying each subject based on his/her compliance of the PA WHO recommendations.

We are aware that our research may have limitations. First, it is a cross-sectional study; therefore, the subjects were not followed over time, and, thus, temporal profiles of the cardiovascular characteristics, CRF exposure, and/or time spent in PA (or PA age-related individual trajectories) remain unknown. Consequently, we cannot examine a “causal” relationships between CPARs and arterial properties. For instance, as we do not know the history of an individual, it is possible that PA might not only induce beneficial age-related changes in cardiovascular properties but also imply that healthier subjects could be more likely to undertake regular PA, especially later in life. On the other hand, within each group, there could be subjects with a highly diverse “past and present” history of PA (inter-individual differences). Although, it has been shown that the impact of PA on cardiovascular health is “dose-dependent,” in this study, we focused on analyzing the impact of WHO PA guideline recommendations, and not on the analysis of the impact of different times/modalities/intensities of PA. Nevertheless, prior studies have demonstrated that the effect of regular and/or high-intensity physical training on several CRFs and/or arterial properties performed during youth disappear relatively soon after physical training cessation (data derived from former/retired athletes) (Majerczak et al., 2019). This observation relativizes the compelling need for having a comprehensive past physical history of a subject.

Second, questionnaires were used as a tool to collect data on PA, despite the fact that there are methods able to evaluate PA more objectively and independently of the operator (e.g., accelerometry). Consequently, the completeness and accuracy of information may have been influenced by how parents and/or subjects perceived the questions. However, it should be noted that other techniques or approaches proposed to assess PA have limitations, particularly in children. As an example, there are controversies regarding (i) where the accelerometer should be placed (wrist vs. waist), (ii) which would be the best functions (equations) to quantify PA intensity, (iii) PA thresholds that should be used to classify PA as light, moderate, or vigorous, (iv) minimum recording time that should be considered representative of PA, and (v) the algorithms (e.g., epoch length, sampling frequency) that should be used to assess PA. Additionally, an activity monitor does not capture some activities (e.g., bicycling). Considering the described controversies, economic cost, and mainly the number of subjects to be evaluated and study design, we opted to assess PA working with questionnaires elaborated based on international recommendations (Gómez-García et al., 2021).

## Conclusions

First, regardless of age, sex, and use of antihyperlipidemic and antidiabetic drugs, CPARs was significantly associated with lower BW and BMI, and better lipid and glucose profile as well as improved kidney function.

Second, the independent association between CPARs and the hemodynamic, structural, and functional characteristics of the arterial system were strongly moderated by age of the subjects. In other words, age modulates the association between CPAR status and arterial variations in a different manner according to the decade of life.

Third, during adult life, as age increases in the subjects, CPAR status was associated with a beneficial hemodynamic profile, which is not related to variations in BP levels but strongly related to lower levels of waveform-derived indexes and left ventricular afterload determinants.

Fourth, in subjects younger than 45–55 y, CPAR status was associated with lower aoBP, baBP, and taBP levels (i.e., the younger the subject, the higher the reduction), and higher “relative contribution” of wave reflections to the pressure waveform, explained by Pf reduction but unchanged Pb levels. Importantly, despite younger individuals with CPAR status showing higher levels of wave reflection indexes, which could represent increasing levels of ventricle afterload, the lower levels of aoBP observed in the same individuals point to the opposite: in the end, what the left ventricle really “sees” during systole is the overall central aoBP.

Finally, our results evidence that it would not be strictly needed to perform an approach that implies simultaneous determination of hemodynamic, structural, and functional arterial characteristics, as the information provided by BP levels and waveform-derived indexes would be enough to evaluate the impact of CPARs on the vascular system in the general population, whatever age group is considered (3–90 y).

## Data Availability Statement

The raw data supporting the conclusions of this article will be made available by the authors, without undue reservation.

## Ethics Statement

The studies involving human participants were reviewed and approved by Comité de Ética del Hospital de Clínicas, Comité de Ética del Centro Hospitalario Pereira-Rossell and Comité de Ética del Instituto Superior de Educación Física (Universidad de la República). Written informed consent to participate in this study was provided by the participant (adult) or participants' legal guardian/next kin (children and adolescents).

## Author Contributions

YZ and DB contributed to the conception and design of the study, and performed the statistical analysis. YZ, MG-G, JT, and DB performed the cardiovascular non-invasive recordings and constructed and organized the database, and wrote the first draft and final version of the manuscript, contributed to manuscript revision, and read and approved the submitted version. All the authors contributed to the article and approved the submitted version.

## Funding

This research was funded by Agencia Nacional de Investigación e Innovación (ANII; PRSCT−008–020), and extra budgetary funds were provided by DB, YZ, and CUiiDARTE Centre.

## Conflict of Interest

The authors declare that the research was conducted in the absence of any commercial or financial relationships that could be construed as a potential conflict of interest.

## Publisher's Note

All claims expressed in this article are solely those of the authors and do not necessarily represent those of their affiliated organizations, or those of the publisher, the editors and the reviewers. Any product that may be evaluated in this article, or claim that may be made by its manufacturer, is not guaranteed or endorsed by the publisher.

## Figures and Tables

**Table:** 

**Abbreviation**	**Definition**
ABI	Ankle-brachial index
AIx	Augmentation index
AIx@75	Heart rate (75 beats/minute) corrected augmentation index
aoBP	Aortic blood pressure
aoDBP	Aortic diastolic blood pressure
aoPP	Aortic pulse pressure
aoSBP	Aortic systolic blood pressure
AP	Augmented pressure
BA	Brachial artery
baDBP	Brachial artery diastolic blood pressure
baSBP	Brachial artery systolic blood pressure
BH	Body height
BP	Blood pressure
BW	Body weight
CCA	Common carotid artery
CFA	Common femoral artery
cfPWV	Carotid-femoral pulse wave velocity
CI	Cardiac index
CO	Cardiac output
CPAR	Compliance with physical activity recommendations
CRFs	Cardiovascular risk factors
crPWV	Carotid-radial pulse wave velocity
CVD	Cardiovascular disease
DD	Arterial diastolic diameter
ECA	External carotid artery
EDV	End diastolic blood flow velocity
EM	Elastic modulus
FMD%	Flow-mediated dilation
HR	Heart rate
ICA	Internal carotid artery
IMT	Intima-media thickness
LFMC%	Low flow-mediated constriction
PA	Physical activity
Pb	backward component of the aortic pulse wave
Pf	forward component of the aortic pulse wave
PI	Doppler-derived pulsatile index
PRV	Peak reversal diastolic velocity
PSV	Peak systolic blood flow velocity
RH	Reactive hyperemia
RIx	Reflection Index
RI	Doppler-derived resistive index
RM	Reflection magnitude
SVR	Systemic vascular resistances
SysD	Arterial systolic diameter
taDBP	Tibial artery diastolic blood pressure
taSBP	Tibial artery systolic blood pressure
TPD_FMD%	Time to peak diameter during flow-mediated dilation
TVR	Total vasoactive range or vascular reactivity
VA	Vertebral artery
VR	Vascular reactivity
β	Stiffness Index
